# Noncoding RNA TY1 reverses fibrosis in systemic sclerosis by attenuating the cGAS/STING pathway

**DOI:** 10.1172/jci.insight.197716

**Published:** 2026-09-08

**Authors:** Xaviar M. Jones, Salwa Soussi, Alessandra Ciullo, Kara Tsi, Weixin Liu, Liang Li, Mario Fournier, Thassio Mesquita, Alberto M. Marchevsky, Nunzio Bottini, Francesco Boin, Ahmed G.E. Ibrahim, Eduardo Marbán

**Affiliations:** 1 Smidt Heart Institute,; 2 Department of Pathology and Laboratory Medicine, and; 3 Kao Autoimmunity Institute and Division of Rheumatology, Department of Medicine, Cedars-Sinai Medical Center, Los Angeles, California, USA.

**Keywords:** Cardiology, Inflammation, Fibrosis, Noncoding RNAs

## Abstract

DNA damage and the cGAS/STING innate immunity pathway have been associated with fibrosis in systemic sclerosis (SSc), but a cause-and-effect role has not been established. Here we report the effects of TY1, a noncoding RNA drug of the exomer class that suppresses DNA damage and thereby inhibits cGAS/STING, in human SSc cells and in 2 preclinical models of SSc. Macrophages from patients with SSc exhibited high levels of phosphorylated DNA damage, cGAS, 2’3’-cGAMP, STING, and IFNs, all of which decreased after exposure to TY1. In mice that had been injected s.c. with bleomycin to model SSc, exercise tolerance, cardiac function, lung hydroxyproline, and skin thickness reverted to normal levels after oral administration of TY1. Similar therapeutic benefits were evident in the genetic *tsk-1* mouse model of SSc. TY1 attenuated fibrosis and/or fibrotic gene expression in both mouse models of SSc and in human SSc skin fibroblasts. Our findings support the hypothesis that cGAS/STING, activated by DNA damage, is a key driver of fibrosis in SSc.

## Abstract 2

We show that noncoding RNA, TY1, reduces inflammation and organ fibrosis in scleroderma, suggesting potential as a novel therapy

## Introduction

Scleroderma is an autoimmune disorder, affecting ~100,000 people in the United States, characterized by fibrosis and tightening of the skin (1, 2). Additional overt involvement of internal organs, a condition known as systemic sclerosis (SSc), portends particularly bad outcomes with a median survival < 10 years (3). Progressive fibrosis of the heart and lungs undermines vital organ function, driving the high mortality. No specific therapeutic options are available. The exact cause of SSc remains unknown. One potential pathogenic potentiator is DNA damage. The cyclic GMP-AMP synthase (cGAS) and stimulator of IFN genes (STING) cascade (cGAS/STING) (4) is a primordial innate immunity pathway that can be turned on by endogenous DNA damage (5), leading to inflammation, tissue damage, and senescence. DNA damage is evident in peripheral blood mononuclear cells (PBMCs) from patients with SSc (6), and the levels of DNA damage correlate with the expression of type I IFN–induced genes that promote tissue fibrosis (7–9). Likewise, hyperactivation of the cGAS/STING pathway has been reported in human SSc skin fibroblasts (10) and in lung tissue and fibroblasts from patients with SSc. The latter study showed cGAS expression to correlate with production of cytokines and type 1 IFNs that were attenuated by G140, a small-molecule cGAS inhibitor that also lessened lung fibrosis in a bleomycin-induced mouse SSc model (11). While the salutary effects of G140 are consistent with a pathogenic role of cGAS/STING activation in SSc, no evidence supports a causal relationship at the level of upstream DNA damage.

Here we probed the role of DNA damage in SSc using TY1, a noncoding RNA drug of the exomer class that upregulates 3′ DNA exonuclease TREX1 and inhibits downstream cGAS/STING (12). Macrophages and fibroblasts from patients with SSc exhibit excess basal activity of cGAS/STING and profibrotic effectors, which are collectively attenuated by TY1. Oral administration of TY1 (13) in 2 complementary mouse models of SSc leads to striking reversal of disease manifestations in skin, heart and lung, with corresponding attenuation of cGAS/STING. Our findings support a causal relationship of cGAS/STING activation, driven by DNA damage, in the pathogenesis of SSc.

## Results

### TY1 attenuates DNA damage and cGAS/STING in human SSc macrophages.

**Figure 1:**
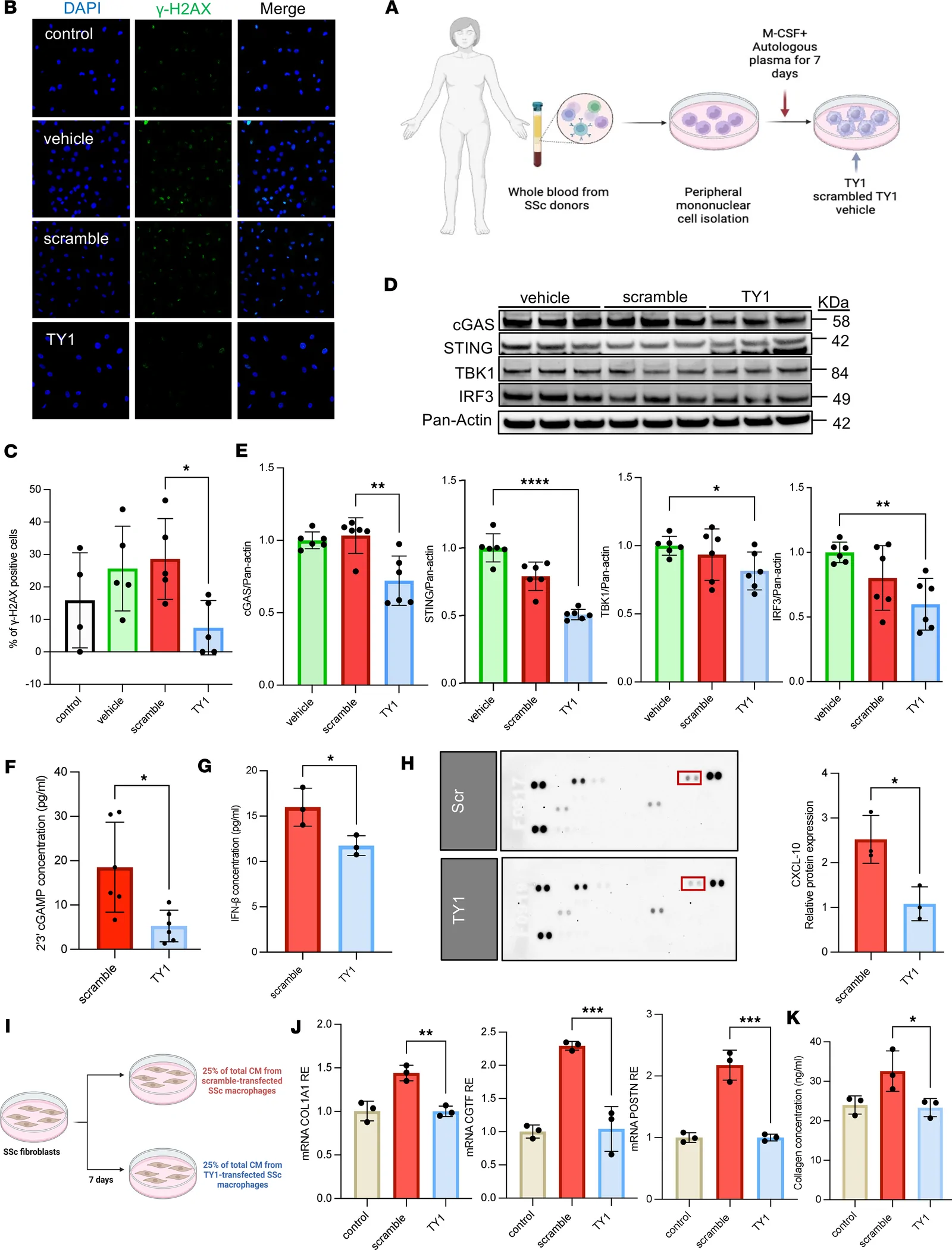
Human SSc macrophages and fibroblasts exhibit attenuation of cGAS-STING and profibrotic cytokines in response to TY1. (**A**) Schematic describing the experimental protocol for human SSc macrophage generation and exposure to vehicle, scramble or TY1 for 24 hours (*n* = 6 biological replicates). Confocal microscopy images of γ-H2AX staining in healthy macrophages or SSc macrophages exposed to Vehicle, Scramble or TY1 (*n* = 5 biological replicates). Magnification ×630. (**B** and **C**) Representative images and pooled data for % of γ-H2AX positive cells. (**D** and **E**) Representative images and analysis of Western blots showing decreases of cGAS, STING, total TBK-1, and total IRF-3 protein levels in SSc macrophages transfected with TY1 compared with vehicle (*n* = 6 biological replicates/group). (**F** and **G**) Quantification of interferon-β and 2’3 cGAMP in conditioned media from scramble- and TY1-transfected SSc macrophages by ELISA (*n* = 6 biological replicates/group). (**H**) Conditioned media from TY1 transfected macrophages show decreased levels of proinflammatory mediators by Proteome Profiler Human Cytokine Array (*n* = 3 biological replicates/group). (**I**) Schematic of the experimental protocol for human SSc fibroblast incubation with diluted SSc macrophage-conditioned media (*n* = 6 biological replicates). (**J** and **K**) Downregulation of mRNA levels of pro-fibrotic markers and secreted collagen in SSc fibroblasts exposed to TY1-transfected SSc macrophages. Statistical analysis by repeated-measures ANOVA followed by Tukey post hoc test with 95% CI; *P < 0.05, **P < 0.01, ***P < 0.001, ****P < 0.0001. Figure created with BioRender (biorender.com) under a Cedars Sinai Medical Center license.

To evaluate DNA damage, we quantified γ-H2AX, a phosphorylated form of the histone protein H2AX, which serves as a sensitive marker for DNA double-stranded breaks in macrophages from healthy human donors (control) or patients with SSc (Supplemental Table 1; supplemental material available online with this article; https://doi.org/10.1172/jci.insight.197716DS1) (14). The experimental protocol to generate human macrophage in culture is depicted in Figure 1A. Compared with control, γ-H2AX was elevated in SSc macrophages exposed to vehicle, while SSc macrophages demonstrated a trend toward increased cGAS/STING signaling compared with healthy donor–derived macrophages (Figure 1, B and C). To probe the role of DNA damage, we used the RNA drug TY1, which enhances the clearance of damaged DNA fragments and attenuates nuclear DNA damage (12). Strikingly, SSc macrophages exposed to TY1 decreased γ-H2AX, reducing it to control levels. To assess whether the effects of TY1 were specific to its sequence, we used a scrambled RNA of the same nucleotide content as TY1 (scramble) but reordered so as to lack any homology to the human or murine genomes. Previous in vitro and in vivo assays have verified the biological inertness of scramble (12). Indeed, transfection with scramble had no effect on the percentage of γ-H2AX^+^ SSc macrophages (Figure 1, B and C). Similarly, TY1, but not scramble, showed upregulation of RNA expression by qPCR in both healthy and SSc macrophages (Supplemental Figure 1, A and B). TY1’s dramatic attenuation of DNA damage is consistent with its known mechanism of action (12), while the inactivity of scramble affirms the specificity of TY1’s benefits.

We next investigated whether the genoprotective effect of TY1 influences downstream cGAS/STING signaling. Cell lysates from human SSc macrophages in primary culture exhibited reduced protein expression of cGAS, STING, TBK1, and IRF-3 after exposure to TY1, relative to scramble or vehicle (Figure 1, D and E). TY1 also decreased the levels of 2′3′-cGAMP and IFN-β in conditioned media (CM) from SSc macrophages (Figure 1, F and G). Inflammatory cytokines such as CXCL-10 are secreted by SSc macrophages, turning on IFN production and contributing to tissue damage and ultimately fibrosis in SSc. In media conditioned by SSc macrophages, CXCL-10 levels were reduced by TY1 exposure (Figure 1H). To test if TY1 requires Trex1 for its therapeutic effects, we knocked down Trex1 using siRNA in SSc macrophages (Supplemental Figure 1, C–E). When *Trex1* was suppressed, TY1 had little effect on fibrosis in SSc fibroblasts, as demonstrated by unchanged gene expression of the profibrotic markers *COL1A1*, *CTGF*, *POSTN*, and *TGFβ* (Supplemental Figure 1D) as well as unchanged collagen production (Supplemental Figure 1E). Collectively, the data demonstrate that TY1 suppresses DNA damage and cGAS/STING signaling by TY1 in human SSc macrophages and leads to downstream tissue repair.

### TY1 prevents fibrotic activation in human fibroblasts.

During SSc progression, tissue resident fibroblasts differentiate into myofibroblasts, which secrete factors stimulating canonical and noncanonical profibrotic pathways (15, 16). Interactions between fibroblasts and macrophages modulate this process (17–20), as exemplified by the profibrotic activation of human cardiac fibroblasts upon exposure to human SSc macrophage CM (21). To dissect macrophage-fibroblast cross-talk, we incubated healthy dermal fibroblasts with TGF-β in the presence of TY1 or vehicle (Supplemental Figure 2A). After 24 hours, fibroblasts exposed to TY1 exhibited lower gene expression of the myofibroblast marker α smooth muscle actin (*αSMA*) and a trend toward lower expression of Collagen 1A1 (*COL1A1*; Supplemental Figure 2B). The same experimental design, applied to human SSc fibroblasts, produced similar results including reduced expression of Collagen 1A2 (*COL1A2*; Supplemental Figure 2C). Previous studies using i.v. and oral delivery demonstrate that TY1 is taken up primarily by macrophages, which, in turn, are necessary and sufficient for TY1’s therapeutic effects (12, 13). To look for possible indirect effects mediated by macrophage-secreted factors, we incubated SSc fibroblasts with media conditioned by SSc macrophages transfected with TY1 or scramble (diluted 75% by volume with conventional fibroblast media; Figure 1I). A separate group of SSc fibroblasts, cultured in conventional fibroblast media, but no CM, served as control. Notably, SSc fibroblasts exposed to CM from TY1-transfected SSc macrophages, but not CM from scramble-transfected SSc macrophages, exhibited decreased expression of profibrotic markers *CTGF*, *POSTN*, and *COL1A1* to transcript levels comparable with those in control. Moreover, SSc fibroblasts exposed to CM from scramble-transfected SSc macrophages expressed elevated col1a1 message levels (Figure 1J). Reductions in levels of profibrotic genes *CGTF* and *POSTN* paralleled the TY1-induced changes in collagen expression (Figure 1J). Collagen secretion was also reduced in fibroblasts directly exposed to TY1 (Figure 1K). These findings support the concept that TY1 prevents myofibroblast differentiation through both direct effects and paracrine immunomodulation by SSc macrophages.

### TY1 attenuates cachexia and fibrosis in bleomycin-injected mice.

**Figure 2:**
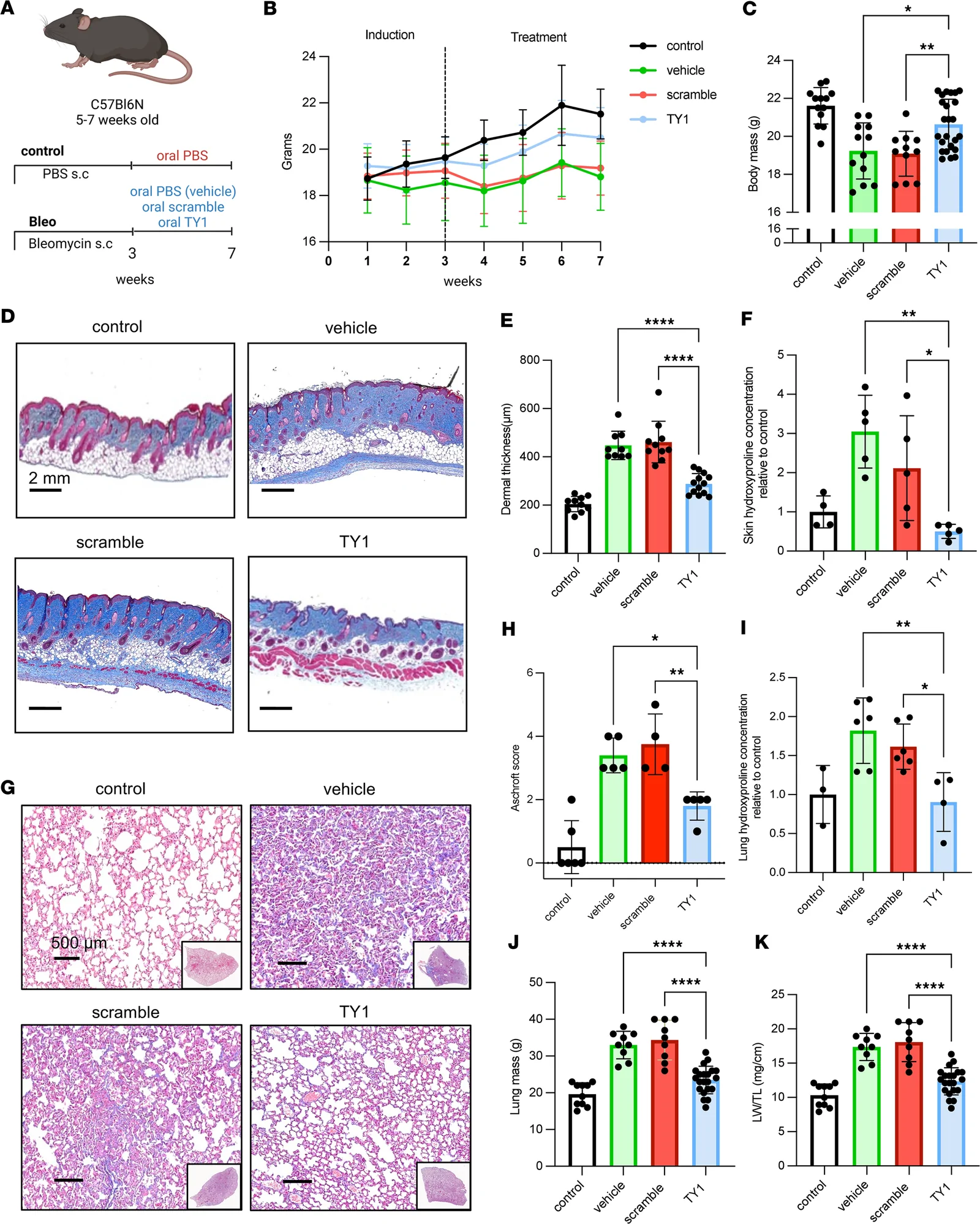
Therapeutic effects of TY1 in mice with skin thickening and SSc-like cardiopulmonary fibrosis after s.c. bleomycin injections. (**A**) Schematic of the experimental protocol. Vehicle-injected (control) or bleomycin-injected mice were fed with vehicle, scramble or TY1 by oral gavage for 4 weeks (control *n* = 10; vehicle *n* = 9; scramble *n* = 9; TY1 *n* = 20). (**B** and **C**) Changes in body mass over time and pooled endpoint data. (**D** and **E**) Representative Masson trichrome images of skin from control mice and bleomycin-injected mice fed vehicle, scramble or TY1, and pooled data for dermal thickness. Scale bar: 2mm. (**F**) Hydroxyproline content in skin tissue (*n* = 4–5 samples /group). (**G**) Representative Masson trichrome images of lung from vehicle- and bleomycin-injected mice fed vehicle, scramble or TY1. Scale bar: 500 µm. (**H**) Analysis of pulmonary fibrosis by Ashcroft score (*n* = 4–6 samples/group). (**I**) Measurement of hydroxyproline content in lung tissue (*n* = 3–6 samples /group). (**J** and **K**) Analysis of lung congestion by lung mass, and lung weight/tibial length. Statistical analysis by repeated-measures ANOVA followed by Tukey post hoc test with 95% CI; *P < 0.05, **P < 0.01, ****P < 0.0001. Figure created with BioRender (biorender.com) under a Cedars Sinai Medical Center license.

If DNA damage and subsequent canonical cGAS/STING activation are pathogenic in SSc, it is logical to predict that TY1 would have disease-modifying bioactivity, as it does in models of myocardial infarction and of heart failure with preserved ejection fraction (12, 22). While no single animal model of SSc reproduces all features of the human disease (23), repeated bleomycin administration causes cachexia, tissue inflammation, and fibrosis of the skin and internal organs, mimicking SSc (24–26). After subdermal bleomycin injections over the course of 3 weeks (at which time the disease phenotype was already evident), mice were randomized to receive vehicle, scramble, or TY1 via oral gavage in casein-chitosan micelles twice weekly for 4 weeks (Figure 2A) (13). Oral administration produced benefits comparable with those of parenteral TY1 (Supplemental Figure 3) and was chosen due to its greater translational relevance. As expected (27), administration of bleomycin led to significant weight loss, but animals fed TY1 showed some weight recovery compared with animals fed vehicle or scramble (Figure 2, B and C). To assess the effects of TY1 on fibrosis, we analyzed lung and skin biopsies from bleomycin-injected mice. Relative to controls, the skin was thickened and fibrotic (histologically and by hydroxyproline content) in bleomycin-injected mice that received vehicle or scramble, but TY1 reversed the skin abnormalities to near-control levels (Figure 2, D–F). Such antifibrotic effects were not confined to skin tissue. Lung samples from TY1 animals also showed less fibrosis (lower Ashcroft score and hydroxyproline content; ref. 28), accompanied by attenuated pulmonary congestion (Figure 2, G–K).

**Figure 3:**
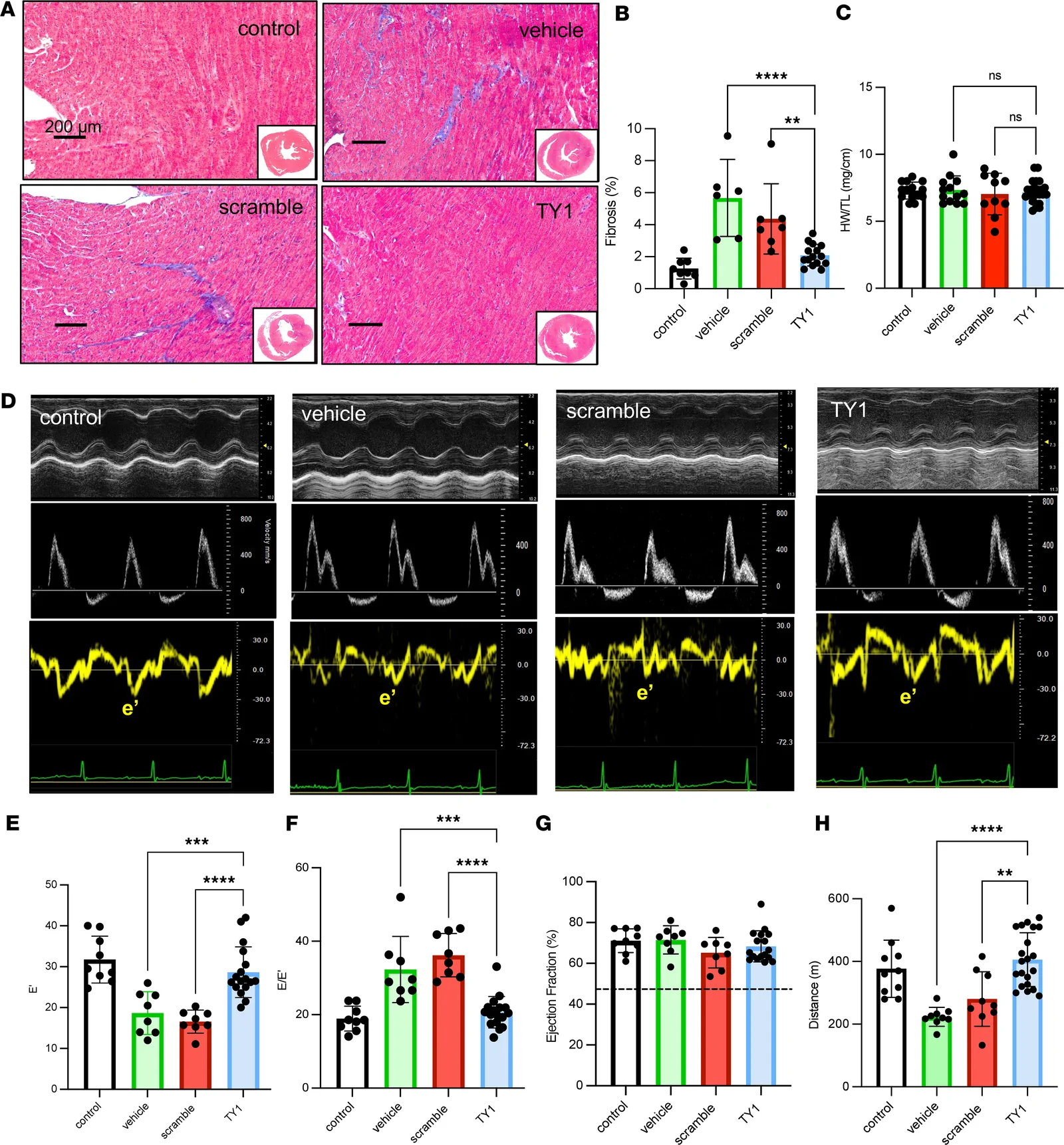
Hearts of mice fed TY1 exhibit antifibrotic effects and attenuation of diastolic dysfunction. (**A** and **B**) Representative Masson trichrome cardiac images from vehicle and bleomycin-injected mice treated with vehicle, scramble or TY1, and pooled analysis of percentage of fibrosis. Scale bar: 200 µm. (**C**) Pooled data for cardiac hypertrophy, measured by heart weight/tibial length. (**D**) Representative echocardiographic M-mode, tissue Doppler, and pulse wave Doppler images. (**E**–**G**) Echocardiographic tissue Doppler early diastolic mitral annular velocity (e’), ratio of transmitral Doppler early filling velocity to tissue Doppler early diastolic mitral annular velocity (E/e’), and ejection fraction (EF, expressed as percent). (**H**) Maximal distance during treadmill exercise (m) by mice in the various experimental groups. Statistical analysis by Student’s *t* test or repeated-measures ANOVA followed by Tukey post hoc test with 95% CI; **P < 0.01, ***P < 0.001, ****P < 0.0001.

Cardiovascular complications are a prominent cause of SSc-related death (29, 30). In particular, cardiac fibrosis and diastolic dysfunction (an early marker of restrictive cardiomyopathy) are diagnosed in 18%–62% of patients with SSc and herald a poor prognosis (31, 32). We (21) and others (33–35) have demonstrated cardiac fibrosis and impaired systolic and diastolic function in bleomycin-induced mice, mimicking the phenotype of patients with SSc with heart involvement. Hearts from TY1-treated mice displayed much less cardiac fibrosis than hearts from mice that had received vehicle or scramble (Figure 3, A and B), without evidence of hypertrophy (Figure 3C). Echocardiography at endpoint revealed diastolic dysfunction in vehicle and scramble groups, but not in TY1 mice (Figure 3D), which exhibited improved e’ and reduced E/e’ ratio (Figure 3, E and F) with preserved ejection fraction (Figure 3G). Furthermore, exercise endurance was impaired in vehicle or scramble groups but restored to control levels in TY1 mice (Figure 3H).

### TY1 prevents skin and cardiac fibrosis in tsk-1 mice.

The bleomycin model of SSc exhibits inflammation-induced fibrosis. To determine if TY1 also modifies disease when fibrotic signaling is less directly coupled to inflammation, we used the tight skin (*tsk-1*) mouse model of SSc, which harbors a mutation in the fibrillin-1 gene, a component of extracellular microfibrils abundant in skin of patients with SSc (36, 37). Homozygous *tsk-1* mice die in utero, while heterozygous littermates show elevated circulating autoantibodies and robust dermal and cardiac fibrosis without overt inflammation — a pattern which resembles advanced fibroproliferative stages of SSc. Accordingly, oral gavage of vehicle, scramble, or TY1 was initiated in 5-week-old heterozygous *tsk-1* mice (Supplemental Figure 4A). Four weeks later, vehicle-fed *tsk-1* mice showed elevated levels of circulating autoantibody Scl-70, as well as prominent skin fibrosis evidenced by increased dermal thickness and hydroxyproline content. TY1 blunted or fully reversed the increases in Scl-70 serum levels (Supplemental Figure 4B), dermal thickness (Supplemental Figure 4, C and D), and skin hydroxyproline content (Supplemental Figure 4E). Cardioprotective effects were also evident: TY1-fed *tsk-1* mice had less cardiac fibrosis (Supplemental Figure 4, F and G) and less hypertrophy than vehicle or scramble mice (Supplemental Figure 4H). Thus, TY1 exerts therapeutic benefits not only in human SSc cells but also in 2 complementary mouse models of SSc, consistent with the hypothesis that DNA damage is a central pathogenic feature that transcends the limitations of any single model.

### TY1 modulates the immune response and attenuates cGAS/STING in bleomycin-injected mice.

**Figure 4:**
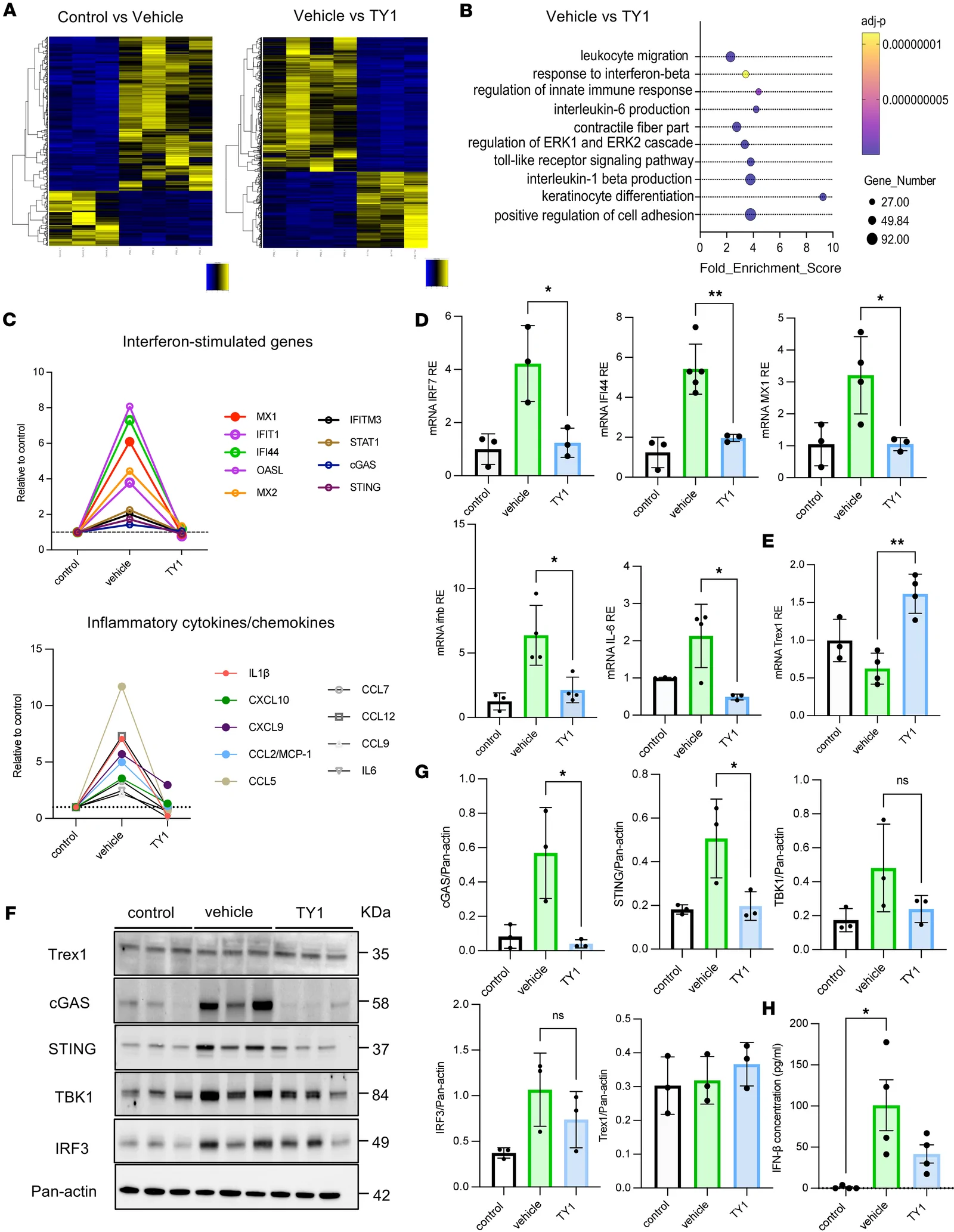
Transcriptomic and protein-level attenuation of cGAS-STING and downstream effectors by TY1 in skin from bleomycin-injected mice. (**A**) Heatmap of transcriptomic sequencing from lesional skin tissue from control and bleomycin-injected mice treated with vehicle or TY1 (*n* = 3–4 biological replicates/group). (**B**) Gene ontology analysis identifies downregulation of DNA damage response, IFN, and inflammatory pathways as major implicated in lesional skin in TY1-treated mice. (**C**) Downregulation of interferon-stimulated and inflammatory genes. (**D**) qPCR validation TY1 inflammatory modulation in skin tissue from control and bleomycin-injected mice treated with vehicle or TY1 (*n* = 3–4 biological replicates/group). (**E**) Trex1 expression in skin tissue from control and bleomycin-injected mice treated with vehicle, scramble, or TY1 by qPCR (*n* = 3–4 biological replicates/group). (**F** and **G**) Representative images and analysis of Western blots showing decreases of cGAS, STING, TBK-1, and IRF-3 protein levels in skin tissue of animals receiving TY1 compared with vehicle (*n* = 3 biological replicates/group). (**H**) Measurement of IFN-β in lesional skin from control and bleomycin-injected mice treated with vehicle or TY1 by ELISA (*n* = 3 biological replicates/group). Statistical analysis by repeated-measures ANOVA followed by Tukey post hoc test with 95% CI; *P < 0.05, **P < 0.01.

To further characterize the benefits of TY1 in SSc, we performed bulk RNA-seq of skin from control (i.e., healthy, no bleomycin), vehicle, and TY1 animals at study endpoint (Figure 4). Heatmaps reveal numerous transcriptional differences between control and vehicle-injected skin, most of which were reversed, in a remarkably coordinated manner, by TY1 (Figure 4A). By gene ontology analysis, pathways related to inflammatory activation were especially attenuated by TY1; these include responses to *IFN-β*, *IL-1*, and *IL-6* production and regulation of the innate immune response (Figure 4B). Genes of the canonical cGAS/STING pathway and the type I IFN response were markedly downregulated in TY1 mice, again in a strikingly coordinated fashion (Figure 4C). Figure 4D shows qPCR confirmation of the pattern of changes in expression of inflammatory genes *IRF7*, *IFI44*, *Mx1*, *IFN-β*, and *IL-6*: upregulation in vehicle samples but attenuation in the TY1 group. In contrast, DNA exonuclease *Trex1* was suppressed in vehicle but upregulated by TY1 (Figure 4E), consistent with the RNA drug’s upstream mechanism of action to suppress DNA damage (12). At the protein level, vehicle mice displayed increased levels of circulating proinflammatory mediators C-reactive protein (CRP), CXCL-2, EGF, IL-7, and LIF, while TY1 blunted these responses (Supplemental Figure 5). To determine whether dysregulated inflammation and skin fibrosis result from activated canonical cGAS/STING activity, we analyzed whole-skin lysates. Protein levels of cGAS and STING, and downstream effectors TBK1 and IRF-3, were elevated in skin samples of vehicle mice compared with controls but reduced to normal levels in the skin of TY1 mice (Figure 4, F and G). TY1 prominently reduced the protein levels of cGAS and STING in skin, while TBK1 and IRF-3 showed nonsignificant trends toward normalization (Figure 4, F and G). In contrast, Trex1 protein levels remain unchanged across groups in this bulk tissue assay, not inconsistent with the concept that upregulation of TREX1 in macrophages underlies the effects of TY1. IFN-β levels were markedly elevated in skin lysates from vehicle mice compared with healthy controls but blunted by TY1 (albeit insignificantly; Figure 4H). Taken together, these data further support the hypothesis that activation of cGAS/STING contributes to skin fibrosis in the bleomycin SSc model. The molecular changes in mice faithfully reproduce, and extend, those seen in human SSc macrophages and fibroblasts exposed to TY1 (Figure 1).

## Discussion

Systemic sclerosis is a complex autoimmune disease characterized by an overactive inflammatory response and internal organ fibrosis, with a 10-year mortality of 30%–50% (3, 38). The exact etiology of SSc remains unknown. Dysregulated immune activation is increasingly recognized as a significant contributor to SSc progression (39). During early stages of SSc, innate and adaptive immune cells promote systemic inflammation and tissue damage through secretion of proinflammatory cytokines, chemokines, and autoantibodies (40–42). Endogenous proinflammatory molecules including cytosolic DNA are released as a result of oxidative stress and cellular injury, and they can bind to TLRs and cGAS. Cytosolic DNA can also activate cGAS and trigger the DNA damage response (6). These events, in turn, kick-start proinflammatory activation with a prominent IFN response, and differentiation of fibroblasts into myofibroblasts (39). Immune targeted therapies (i.e., rituximab) have shown some benefits in SSc-associated skin and lung fibrosis but no mortality benefit (43–45). Here, we provide further evidence of the pivotal roles of hyperinflammation and IFN signaling in scleroderma and SSc. Chronic oral administration of TY1 improves physical function of the heart and body and reverses fibrosis in 2 mouse models (bleomycin-induced and Tsk-1 transgenic) with active, progressive SSc phenotypes. DNA damage has been associated with SSc but not necessarily in a cause-and-effect relationship (6). The fact that TY1 — which modulates TREX1, an enzyme that degrades damaged DNA — has such dramatic therapeutic benefits is consistent with the hypothesis that DNA damage is central to SSc pathogenesis. A scramble RNA has no effects, verifying that the benefits are specific to the TY1 sequence and not mimicked by another RNA of the same size and nucleotide composition.

This study has limitations. We did not systematically include healthy donor-derived macrophages across all signaling assays, which would further clarify the extent to which TY1 restores physiological baseline activity. Meanwhile, although TY1 increased *Trex1* transcript levels, we did not observe a corresponding increase in total Trex1 protein in whole tissue lysates. While in vitro studies establish a mechanistic link between DNA damage reduction and suppression of cGAS/STING–associated signaling pathway, further studies are needed to confirm the mechanism in vivo. Such discrepancy may be explained by cell type–specific effects, as observed in the macrophage response after TY1 treatment (Supplemental Figure 1, A and B). Future studies incorporating single-cell resolution, and animal models with cell-specific genetic perturbations will be required to fully delineate mechanisms underlying TY1’s antifibrotic effect. Nevertheless, having linked DNA damage to canonical cGAS/STING activation in SSc, Paul et al. (10) presciently proposed that inhibition of this cascade could be a game changer for SSc therapeutics. Our findings with TY1 provide experimental verification of their prediction. Importantly from a practical perspective, TY1 does not just prevent disease progression; it actually reverses established disease, something exceptional in SSc therapeutics.

In the last decade, numerous preclinical and clinical studies have found marked overexpression of IFN response genes (also referred to as “IFN signature”) in patients with rheumatic diseases (46, 47). Microarray analysis of blood samples from lupus or patients with SSc revealed a homology in > 90% of abnormal IFN-inducible genes, which correlated with disease activity (48). However, the origin of the IFN response remains elusive. In the context of autoimmune diseases, genotoxic stress (secondary to chemical exposure, ROS, genetic or epigenetic factors) figures as a leading cause of DNA breakage, which can alter cellular function and trigger nucleic acid recognition mechanisms (including the cGAS/STING pathway) and downstream inflammatory mediators. Furthermore, loss of function mutations in TREX1 have been linked to autoimmunity (49–51). Collectively, these results highlight the role of cGAS/STING activation in SSc pathogenesis. Micronuclei (i.e., cytosolic DNA aggregates) are formed in SSc fibroblasts, with cGAS/STING upregulation and inflammatory responses (10). We found the cGAS/STING pathway to be overactive in an animal model of SSc and in human SSc macrophages. TY1 diminished cGAS/STING, 2’3’cGAMP, and IFN response molecules (IFN and CXCL-10) coordinately. Targeting macrophages influences other cell types, insofar as media conditioned by TY1-transfected macrophages prevented profibrotic activation and collagen secretion in SSc fibroblasts.

Even more than systemic inflammation, tissue fibrosis is the main contributor to the elevated mortality in SSc (29). TY1 attenuated fibrosis in both bleomycin-induced and *tsk-1* mouse models. While the former model mimics early, inflammatory stages of SSc, the latter represents the advanced fibroproliferative phenotype of the disease. It is remarkable that TY1 demonstrated antifibrotic efficacy in both models, even when the treatment was initiated after fibrosis had already become established. While modulation of the cGAS/STING pathway underlies TY1’s antifibrotic effects in the bleomycin model, we have yet to fully dissect the mechanism of TY1’s disease-modifying bioactivity in *tsk-1* mice. Such mice are difficult to breed (52), making detailed mechanistic dissection a challenge in this strain. Macrophages may mediate the benefits of TY1 in *tsk-1* mice, as has been proposed for the immunomodulatory drug paquinimod in this model (53), but for now, this remains a conjecture.

Targeting the immune compartment in SSc is hardly a new idea, as evidenced by trials of tocilizumab, rituximab, autologous hematopoietic stem cell transplantation (HSCT), and CAR-T cell therapy (44, 54–59). Among these, HSCT therapy has shown impressive benefits in 2 randomized clinical trials, but at the expense of life-threatening adverse events, limiting real-world applicability. Nevertheless, the macrophage compartment, and innate immunity in general, have not been studied extensively in SSc therapeutics, nor has DNA damage been a specific focus. The findings reported here support testing of TY1 as a potentially novel therapeutic candidate in SSc. TY1 is the index compound in a new class of RNA drugs known as exomers (12), based on ncRNAs mined from extracelluar vesicles. Extensive data (not shown here) support the safety and tolerability of oral TY1. Nevertheless, several RNA drugs have failed in translation due to unforeseen adverse effects in human studies (60); clinical trials will be required to see if TY1 can fulfill its therapeutic promise in this devastating orphan condition.

## Methods

### Sex as a biological variable.

Sex as a biological variable was not systematically explored in this study. All bleomycin-exposed mice were purposely chosen as females, given the fact that 80%–90% of patients with SSc are female. In the Tsk1 mouse model, both sexes were used due to difficulties with breeding, a known feature of this model (61).

### Experimental resources.

Supplemental Table 2 lists relevant facts for all commerical products.

### Animal studies.

We used 2 complementary models of SSc: (a) s.c. bleomycin in WT mice and (b) *Tsk-1* mice. To evaluate the effects of TY1 on bleomycin-induced fibrosis in vivo, 5- to 7-week-old C57BL/6 female mice (Jackson Lab) were injected with 100 μL of bleomycin s.c. (1 mg/mL dissolved in 0.9% NaCl; Cayman) in a single location on the upper back every other day for 3 weeks. Afterward, mice were randomized to receive vehicle, TY1, or scramble (an RNA of the same nucleotide content as TY1), but in scrambled order (12) via oral gavage twice weekly for 4 weeks. A separate group of WT C57BL/6 female mice of matching age and sex were injected 100 μL of 0.9% NaCl s.c. for 3 weeks followed by 4 weeks of vehicle (0.9% NaCl), and served as controls. After a total of 7 weeks, mice were sacrificed by cervical dislocation.

In the *tsk-1 (*Jackson Lab) mouse model of SSc, 5- to 7-week-old heterozygous male and female mice were randomized to receive vehicle, scramble, or TY1 via oral gavage twice weekly for 4 weeks. WT homozygous mice served as healthy controls.

### Synthesis and formulation of RNA compounds.

TY1 is a 24-nucleotide small RNA with 6 locked nucleic acid residues in its backbone. An RNA oligonucleotide of the same nucleotide content, but in scrambled order with no homology to the murine or human genomes, served as a useful control for nonspecific RNA effects (9). TY1 and scramble were manufactured by solid phase synthesis at research grade (IDT). The i.v. formulation consisted of mixing TY1 (or scramble) with transfection reagent (Dharmafect; Horizon Discovery), diluted in 0.9% NaCl to reach a final volume of 100 μL and incubated for 15 minutes at room temperature with gentle agitation. The solution was administered via retro-orbital injection following anesthesia induction in mice. For the oral formulation, TY1-IV solution (0.15 mg/kg) in lipid nanoparticles (Dharmafect), were further encapsulated in casein-chitosan (C2) micelles. To generate C2 micelles, TY1-IV solution was mixed with a 5% casein solution from bovine milk (125 μL per mouse, C4765, Millipore Sigma) and incubated for 15 minutes at room temperature with gentle agitation. Following incubation, 100 μL of a 0.1% acetic acid solution (695092, Sigma-Aldrich)/0.2% chitosan (448869, Millipore Sigma) was added dropwise and mixed well. After 60 minutes of incubation at room temperature, the solution was administered via oral gavage (01-290-3B, Thermo Fisher Scientific); the animal was held with the body tilted upward, and the gavage catheter was inserted into the mouth gently. For macrophage transfection studies, TY1-IV was added to a final concentration of 80 nM. The same process is repeated with scramble.

### Hydroxyproline assay.

Total collagen content of skin and lung tissue samples was quantified using a hydroxyproline assay kit (Millipore Sigma). Briefly, 5N HCl solution was added to skin, lung and heart tissue, followed by hydrolysis at 120°C for 3 hours. The processed tissue samples were incubated with chloramine-T solution for 5 minutes at RT. Each sample was treated with DMAB solution and incubated for 90 minutes at 60°C for color development. Samples were read in a 96-well plate at 560 nm on a multiple microplate reader (Bio-Tek Instruments Inc.).

### Histology.

Lesional skin biopsies from bleomycin- or PBS-injected sites, as well as hearts and lungs, were collected. Paraffin-embedded sections (5 μm) were stained with Masson trichrome or Picrosirius Red and imaged using an Aperio AT Turbo slide scanner (Leica) at ×40 magnification. Cardiac fibrosis was quantified (ImageJ, NIH) as the percent area of blue staining divided by the area of red staining of the entire Masson trichrome tissue section. Dermal thickness was measured from the dermal-epidermal boundary to the hypodermis at 5 randomly selected sites in each mouse and analyzed in a blinded manner. To assess pulmonary fibrosis in samples, the Ashcroft score was analyzed by a pulmonary pathologist in a blinded manner.

### Echocardiography.

Two-dimensional transthoracic echocardiography was performed (Vevo 3100, Visual Sonics) under light isoflurane anesthesia. Parasternal short-axis B-mode videos were recorded, and left ventricular ejection fraction was measured (Visual Sonics v2.0.0 software). Diastolic function was assessed from the apical 4-chamber view by measuring E/e’ ratio. E wave (early filling) was measured by pulse-wave Doppler mode between the tips of the mitral valve; e’ was measured with tissue Doppler mode at the septal corner of the mitral annulus. Three separate measurements from each animal were averaged for each parameter.

### Treadmill.

Mice were placed inside an Exer-3/6 rodent treadmill (Columbus Instruments) at a 5-degree elevation. Animals were acclimated to the device by leaving them undisturbed for 30 minutes and then engaging the belt at a slow pace (10 m/min) for 20 minutes. After the acclimation period was complete, the exercise protocol began; shock grid activated (0.15 mA, 1Hz) and belt speed increased (1 m/min after each minute of exercise). Mice resting on the shock grid for > 10 seconds reached maximal exercise capacity and were removed from the device.

### Protein extraction and isolation.

Skin tissue was collected, rinsed in PBS, and then stored at –80°C until use. Samples were minced, suspended in T-PER Buffer (containing Halt protease and phosphatase inhibitor, Thermo Fisher), homogenized (Bead Ruptor, OMNI), and then centrifuged centrifuged at 16,000 g for 15 minutes. Protein supernatants were collected, and concentrations measured (BCA Protein Assay Kit, Pierce).

### Western blots.

Protein samples were prepared for gel electrophoresis (NuPAGE 4%–12% Bis-Tris, Invitrogen) according to the manufacturer’s protocol. A normalized value between 10 μg and 30 μg was used for loading in each well. Proteins were then transferred to a Nitrocellulose Membrane, 0.45 mm (Thermo Fisher Scientific) for immunoblotting with antibodies. Bands were detected (ChemiDoc Imaging System, Bio-Rad) with ECL Western Blotting Substrate (Pierce).

### SSc patient fibroblasts and monocytes.

Plasma and PBMCs were isolated from whole blood from patients with SSc (*n* = 3) seeking treatment at Cedars-Sinai Scleroderma Clinic. Anonymized dermal fibroblasts from patients with SSc were obtained from the UCSD (62). Cell culture experiments were conducted between cell passage 3 and 7, respectively. All patients with SSc included in this study fulfilled the American College of Rheumatology (ACR)/European League Against Rheumatism classification criteria for SSc (63). All patients were females, between ages 19 and 71 years old and were categorized according to the disease stage with diffuse cutaneous SSc (dcSSc) or limited cutaneous SSc (lcSSc). Patient and control characteristics are represented in Supplemental Table 1. Experimenters were blinded to patient health status.

### Cell culture experiments.

Plasma and PBMCs were obtained from whole blood using Ficoll-Paque Premium (density 1.077; GE Healthcare). To generate SSc or healthy donor–derived macrophages, CD14^+^ monocytes were cultured in complete HEPES-buffered RPMI 1640 (2.05 mM L-glutamine) supplemented with 10% autologous or allogeneic plasma, 50 ng/mL macrophage colony-stimulating factor (M-CSF), and 10 μg/mL gentamicin for 5–7 days (14). To evaluate TY1 effect on macrophages, SSc or healthy donor-derived (nMacs) were plated at 1 × 10^6^ cells/well in 6-well culture dishes in complete RPMI and transfected with scramble or TY1 (80 nM, based on prior dose optimization of EV-YF1 formulated in DharmaFECT) for 24 hours. For studies TREX1 knockdown studies, macrophages were transfected with siRNA against Trex1 (si-TREX1; 80 nM, OriGene) formulated in DharmaFECT for 24 hours. Afterward, macrophages were washed with PBS and cultured in FBS medium for 24 hours. Conditioned medium was collected and centrifuged at 300*g* for 5 minutes to eliminate cell debris, and it was then condensed by an ultrafiltration membrane at 3 kDa. The medium was stored at –80°C until needed.

### Cytokines, 2’3’ cGAMP and IFN-β measurement in mouse skin and cell culture supernatants by ELISA.

For measuring protein levels in mouse skin after bleomycin stimulation, the harvested skins were washed once with cold PBS, transferred into 2 mL tubes, rapidly frozen into liquid-N_2_, and stored at –80°C. Later, to prepare tissue homogenates, tissue protein extraction reagent (T-PER) (Thermo Fisher Scientific) was added and homogenized by a BeadBeater (BioSpec). The lysates were transferred to a 1.5 mL tube and spun at 12,000 rpm for 20 minutes at 4°C. Supernatant was collected for the ELISA measurement according to provider’s protocol. 2’3’ cGAMP and IFN-β in supernatant of SSc macrophages culture and skin homogenates were measured with the ELISA kit (R&D Systems). Cytokines in skin lysates and homogenates and in supernatants of TY1-transfected SSc macrophages cell cultures were analyzed with ELISA kit (R&D Systems). To account for differences in protein concentration between samples, 50 μg of protein were loaded to ELISA experiments. Arrays were detected (ChemiDoc Imaging System, Bio-Rad) following the addition of detection substrate.

### CM experiments on SSc fibroblasts.

To study paracrine effects on fibroblast activation, SSc fibroblasts were plated at 7 × 10^4^ cells/well and supplemented with Dulbecco’s Modified Eagle Medium (DMEM) 1% FBS (75% of total media volume/well), and CM from SSc macrophages transfected with scramble or TY1 (25% of total media volume/well) for 7 days. A separate group of SSc fibroblasts, supplemented with DMEM 1% FBS and unexposed to CM, served as controls. CM was replaced every 2–3 days. For all in vitro experiments, cells were used between passages 3–7 (2–3 biological replicates/group).

### Collagen measurement in SSc fibroblast culture supernatants by ELISA.

Type I collagen in SSc fibroblast CM was measured with the ELISA kit (Abcam) following the manufacturer’s protocol.

### qPCR.

Total RNA was isolated from skin tissue using RNeasy Plus Universal Mini Kit (Qiagen) and cDNA was synthesized with 1 μg of RNA using High-Capacity RNA-to-cDNA Kit (Applied Biosystems). The reaction was performed in QuantStudio 12K Flex Real-Time PCR System, and each reaction was performed in triplicate samples and adjusted using hprt1. The 2^–ΔΔCt^ relative quantification method, using Ldha (Rn00820751_g1) for normalization. Fold change was calculated with 2^–ΔΔCt^ compared with the control group. Log scale of fold change was used to compare expression levels.

### RNA-seq.

Skin RNA samples were sequenced at the Cedars-Sinai Genomics Core as described (64). Total RNA samples were assessed for concentration using a Qubit fluorometer (Thermo Fisher Scientific) and for quality using the 2100 Bioanalyzer (Agilent Technologies). Library construction was performed using the QIASeq Stranded RNA Library kit (Qiagen) with QIAseq FastSelect — rRNA HMR Kit (Qiagen) for ribosomal RNA depletion. Library concentration was measured with a Qubit fluorometer and library size on a Bioanalyzer. Libraries were multiplexed and sequenced on a NovaSeq 6000 (Illumina) using 75 bp single-end sequencing. On average, ~50 million reads were generated from each sample.

### Statistics.

Statistical parameters including the number of samples (*n*), descriptive statistics (mean ± SD), and significance are reported in the figures and figure legends. Differences between groups were examined for statistical significance using the Student’s 2-tailed *t* tests or 1-way ANOVA with Tukey’s post hoc test. Differences with *P* < 0.05 were considered significant.

### Study approval.

The animal experiments followed the *Guide for the Care and Use of Laboratory Animals* (National Academies Press, 2011), and they were approved by the Cedars-Sinai Medical Center Institute Animal Care and Use Committee. For human studies, ethics approval was provided by Cedars-Sinai IRB, and all participants provided their written informed consent.

### Data availability.

All data generated during this study are included in this published article and the Supporting Data Values file. RNA-Seq data are available through the NCBI with accession code PRJNA1476349. Additional detailed information is available from the corresponding author upon request.

## Author contributions

XMJ, AGEI, and EM designed the research. XMJ, SS, AC, KT, WL, LL, MF, and TM performed, analyzed, and interpreted the research. AMM performed the Ashcroft score on lung samples. FB and NB provided essential material. XMJ, AGEI, and EM wrote the manuscript.

## Conflict of interest

EM owns founder’s equity in Capricor. Capricor has no relationship with this project, nor any licensing rights to the discoveries reported here.

## Funding support

This work is the result of NIH funding, in whole or in part, and is subject to the NIH Public Access Policy. Through acceptance of this federal funding, the NIH has been given a right to make the work publicly available in PubMed Central.

- NIH (R01HL164588 to EM and T32HL116273 to EM, which supported XMJ)
- California Institute for Regenerative Medicine (TRAN1-15317 to AGI)

## Supplementary Material

Supplemental data

Unedited blot and gel images

Supporting data values

## Version 1 09/08/2026

Electronic publication
