# Supplementary material for: Noncoding RNA TY1 reverses fibrosis in systemic sclerosis by attenuating the cGAS/STING pathway
Source: JCI Insight. 2026 Sep 8;11(17):e197716. doi: 10.1172/jci.insight.197716 (PMC13564078; doi:10.1172/jci.insight.197716)

SUPPLEMENTARY MATERIAL

"Noncoding RNA TY1 reverses fibrosis in systemic sclerosis by attenuating the cGAS-STING pathway"

Table 1. Patient characteristics

Blood samples used for monocyte isolation, differentiation, and RNA testing

Patient ID	Age at onset	Sex	Race	Phenotype	Immunosuppressants
1	37	Female	White	Diffuse SSc	No
2	45	Female	White	Diffuse SSc	No
3	58	Female	White	Diffuse SSc	No
170	38	Female	Asian	Diffuse SSc	Yes
079	71	Female	White	Limited SSc	Yes
116	42	Male	White	Limited SSc	No

SSc dermal fibroblasts used in SSc-macrophage condition media experiments

Patient ID	Age at onset	Sex	Race	Phenotype	Immunosuppressants
037	38	Female	White	Diffuse SSc	No
069	62	Female	White	Limited SSc	No
079	50	Female	White	Diffuse SSc	No

Table 2. Resource Table

Antibody list			
**Target antigen**	**Vendor or source**	**Catalog #**	**Working concentration**
Trex1 (Mouse)	Cell signaling	76892	1:1000
cGAS (Mouse)	Abcam	ab252416	1:1000
cGAS (Human)	Abcam	ab224144	1:1000
STING (Mouse)	Abcam	ab288157	1:1000
STING(Human)	R&D Systems	AF6516	1:1000
TBK1/NAK	Cell Signaling	3013	1:1000
IRF-3	Cell Signaling	4302	1:1000
Pan-actin	Cell Signaling	8456	1:1000

Product name	Vendor	Catalog #
Bleomycin sulfate	Cayman Chemical	13877
Hydroxyproline assay	Sigma Aldrich	MAK008-1KT
Proteome Profiler Mouse Cytokine Array Kit, Panel A	R&D Systems	ARY006
Proteome Profiler Human Cytokine Array Kit	R&D Systems	ARY005B
Mouse IFN-beta Quantikine ELISA Kit	R&D Systems	MIFNB0
Human IFN-beta ELISA Kit	R&D Systems	41410-1
Human type I collagen ELISA Kit	Abcam	ab285250
2’3’-cyclic GAMP ELISA Kit (Mouse)	Invitrogen	EIAGAMP
2’3’-cyclic GAMP ELISA Kit (Human)	Cayman Chemical	501700
Mouse Scl-70 Antibody ELISA Kit	MyBioSource	MBS726620
RNeasy Plus Universal Mini Kit	Qiagen	73404
High-Capacity RNA-to-cDNA Kit	Applied Biosystems	4388950
Halt™ Protease and Phosphatase Inhibitor	Thermo Scientific	78442
RIPA Lysis and Extraction Buffer	Thermo Scientific	89901
Taqman (Mx1; mouse)	Thermo Scientific	Mm00487796_m1
Taqman (Trex1; mouse)	Thermo Scientific	Mm00810120_s1
Taqman (IRF7; mouse)	Thermo Scientific	Mm00516788_m1

Taqman (IL-6; mouse)	Thermo Scientific	Mm00446190_m1
Taqman (IFI44; mouse)	Thermo Scientific	Mm01320795_m1
Taqman (ifnb;mouse)	Thermo Scientific	Mm00439552_s1
Taqman(HPRT1;mouse)	Thermo Scientific	Mm03024075_m1
Taqman (Trex1; human)	Thermo Scientific	Hs03989617_s1
Taqman(COL1A1;human)	Thermo Scientific	Hs00164004_m1
Taqman(CTGF;human)	Thermo Scientific	Hs00170014_m1
Taqman(POSTN;human)	Thermo Scientific	Hs01566750_m1
Taqman(HPRT1;human)	Thermo Scientific	Hs02800695_m1

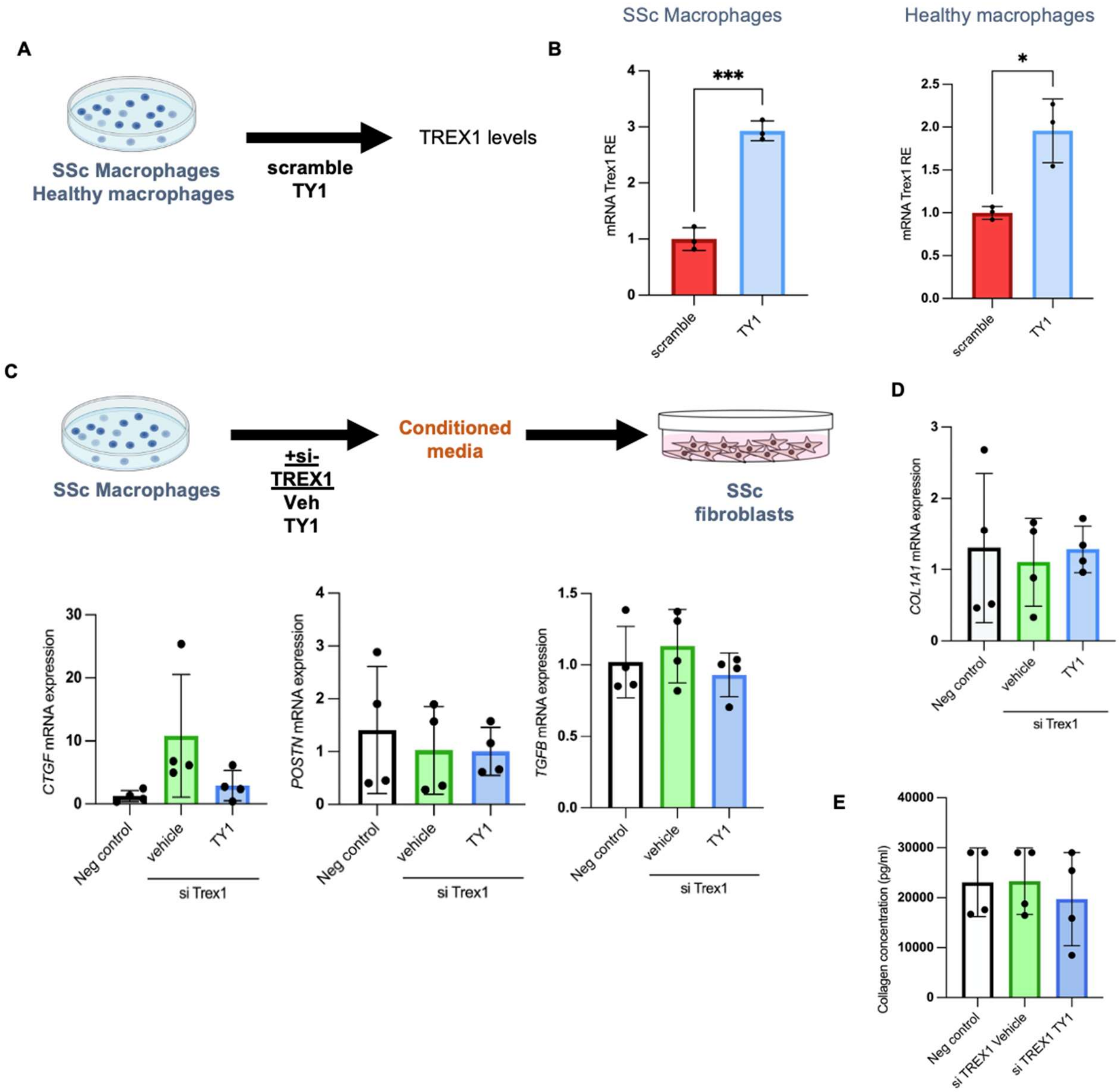

Supplementary Figure 1. Trex1-dependent TY1 immunomodulatory activity on human macrophages and fibroblasts A. Schematic graph of experimental study on healthy and SSc-derived macrophages exposed to TY1 or scramble. Confocal microscopy images of γ-H2AX staining in healthy macrophages or SSc macrophages exposed to Vehicle, Scramble or TY1 (n=5 biological replicates). **B.** Analysis of Trex1 fold change in healthy and SSc macrophages post TY1 and scramble transfection by qPCR (n=3 samples/group). **C.** Schematic of the experimental protocol for human SSc fibroblast incubation with diluted conditioned media from SSc-macrophages transfected with either TY1 or scramble in presence of Trex1 silencing RNA or negative control (n=4 biological replicates). **D.** mRNA levels of pro-fibrotic markers, and **E.** secreted collagen in SSc fibroblasts exposed to TY1-transfected SSc macrophages in presence of Trex1 silencing RNA showing blunted effect. Statistical analysis by repeated measures ANOVA followed by Tukey post hoc test with 95% CI; $^{*}p<0.05$; $^{**}p<0.01$, $^{***}p<0.001$; $^{****}p < 0.0001$.

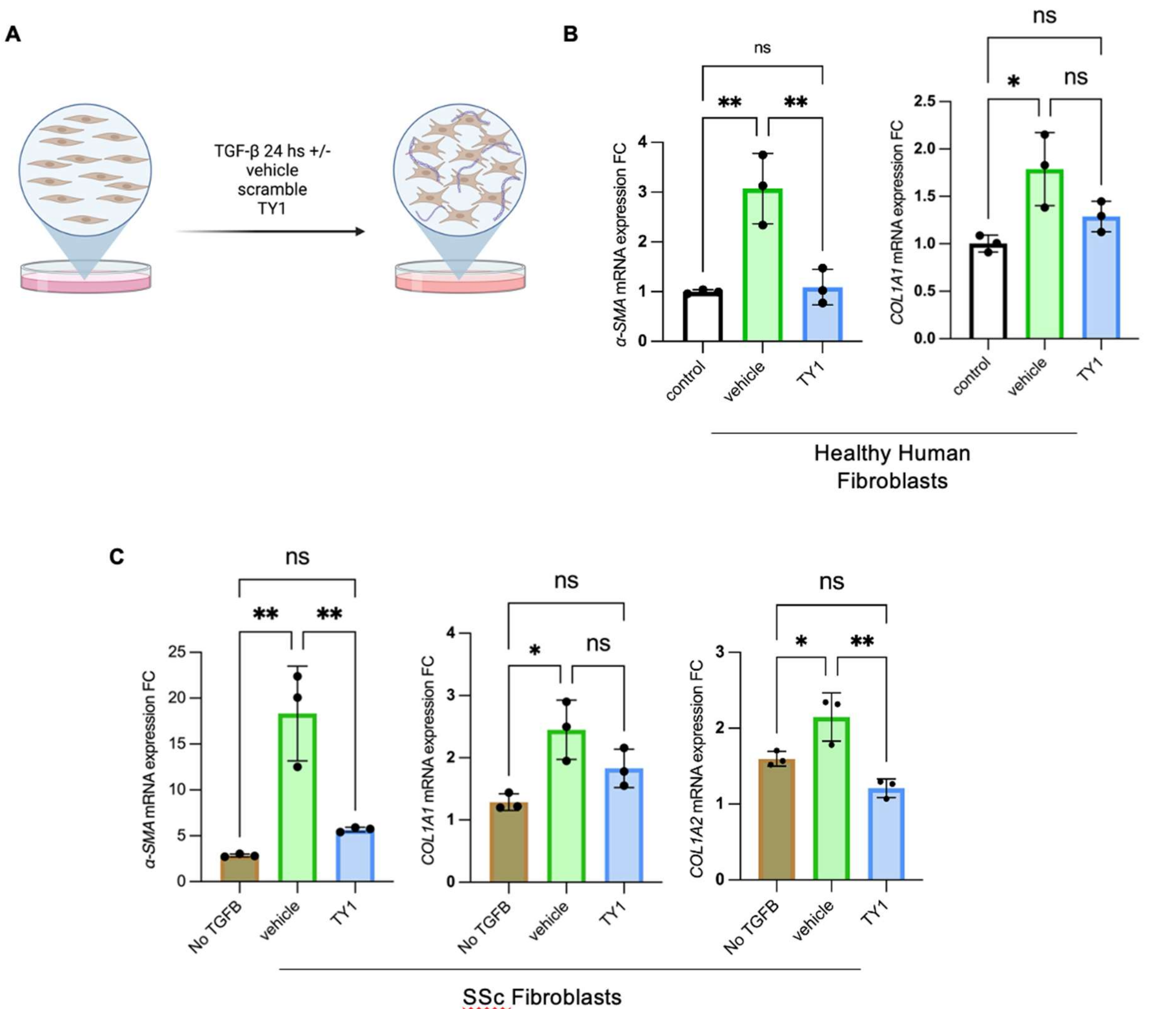

Supplementary Figure 2. Antifibrotic effect of TY1 on healthy and SSc fibroblasts. A. Schematic for pilot study of SSc and healthy fibroblast transfection of vehicle or TY1 plus TGF-β for 24h (3 biological samples per group). **B.** Fold change mRNA expression of fibrotic markers in healthy, and **C.** SSc fibroblasts. Statistical analysis by repeated measures ANOVA followed by Tukey post hoc test with 95% CI; *p<0.05; **p<0.05

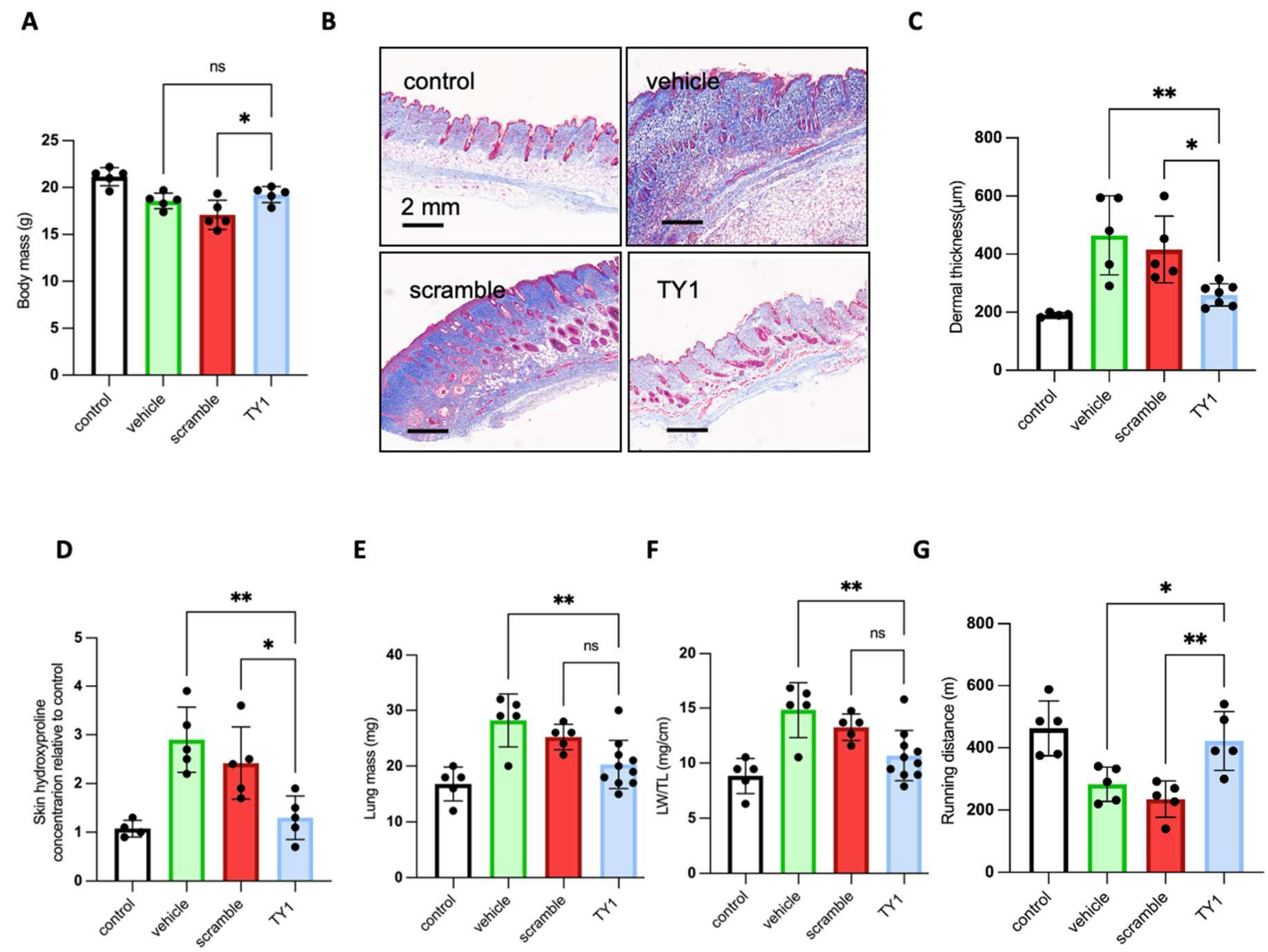

Supplementary Figure 3. Therapeutic effects of intravenous TY1 in bleomycin mice are comparable to those of oral TY1 (cf. Fig 2). A. Body mass at endpoint (n=5 mice/group)**. B.** Representative Masson trichrome images of skin, and **C.** pooled data for dermal thickness**. D.** Measurement of hydroxyproline concentration in skin. **E.** Analysis of lung congestion by lung mass (mg), and **F.** lung weight/tibial length (LW/TL). **G.** Maximal distance covered before exhaustion in a treadmill assay (m). Statistical analysis by repeated measures ANOVA followed by Tukey post hoc test with 95% CI; $^*p<0.05$; $^{**}p<0.01$, $^{***}p<0.001$; $^{****}p < 0.0001$.

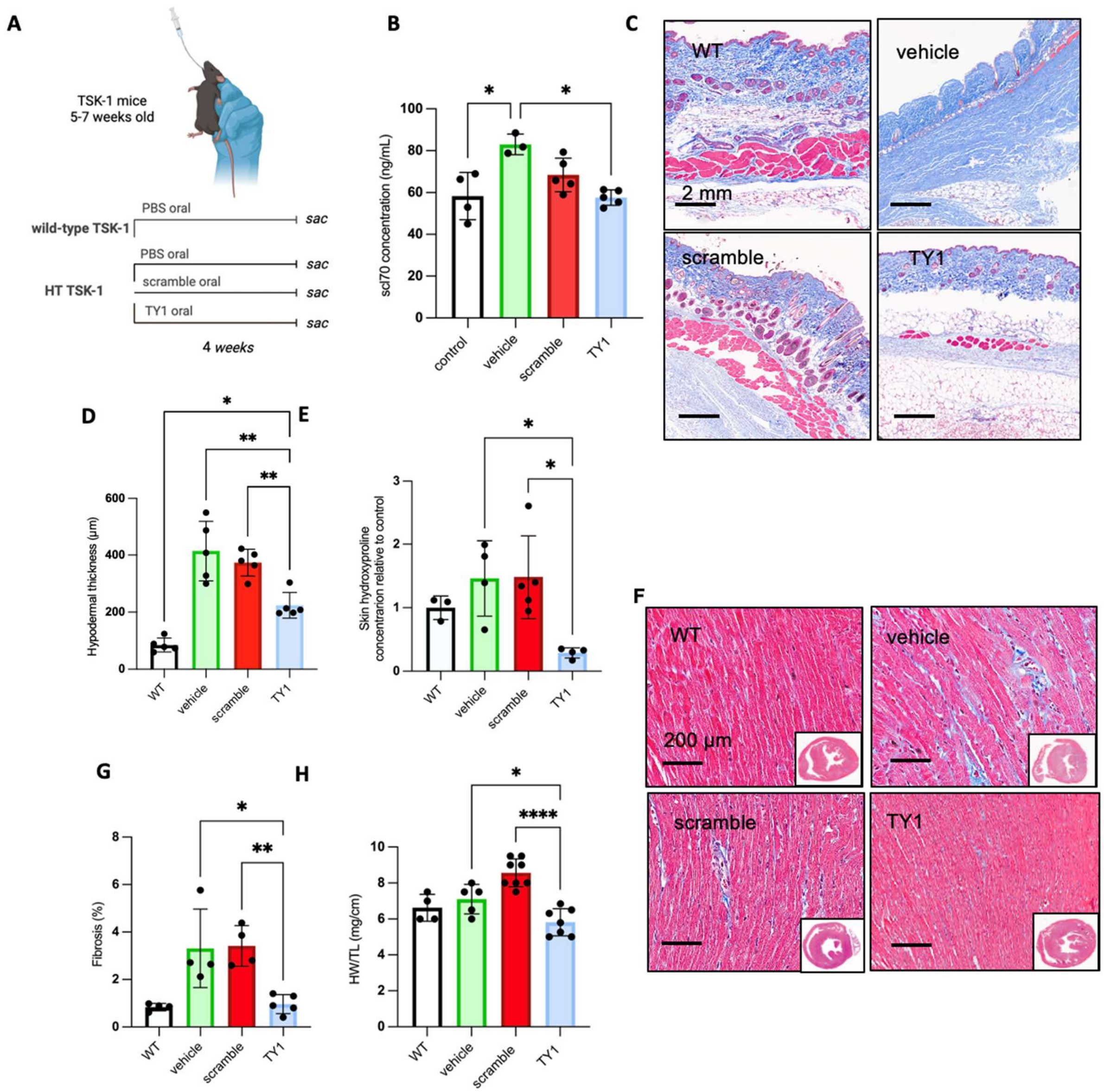

Supplementary Figure 4. Oral TY1 attenuates cutaneous and cardiac fibrosis in Tsk-1 mice. A. Schematic of experimental protocol for wild-type (control) or heterozygous tight skin1 (Tsk-1) mice fed vehicle, scramble or TY1 by oral gavage twice a week (n=4-8 mice/group). **B.** Pooled data for circulating levels of Scl-70 autoantibodies (n=3-5 samples/group). **C.** Representative Masson trichrome images of skin, and **D.** and pooled data for hypodermal thickness. **E.** Measurement of hydroxyproline content in skin tissue (n=3-5 samples /group) **F.** Representative Masson's trichrome cardiac images from vehicle and bleomycin-injected mice treated with vehicle, scramble or TY1, and **G.** pooled analysis of % fibrosis. **H.** Pooled data for heart weight/tibial length. Statistical analysis by repeated measures ANOVA followed by Tukey post hoc test with 95% CI; *p<0.05; **p<0.01, ***p<0.001.

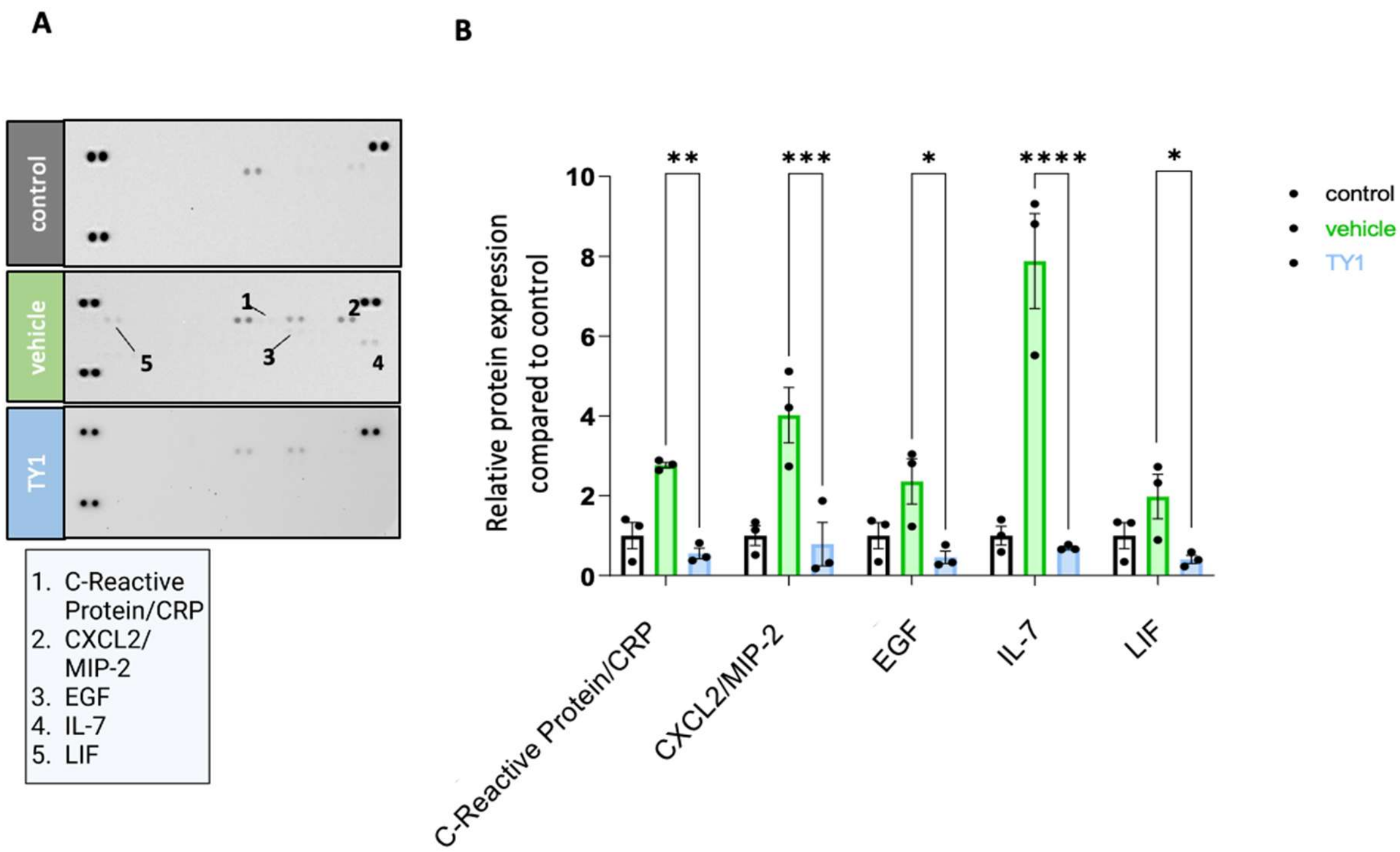

Supplementary Figure 5. TY1-fed mice exhibit reduced cytokine levels in skin tissue samples. A. Representative image of membrane arrays used for profiling cytokines and chemokines in skin samples from control, vehicle and TY1 mice (n=3 biological replicates/groups). **B.** Relative expression of cytokines and chemokines in each group, normalized to levels in control mice. Statistical analysis by repeated measures ANOVA followed by Tukey post hoc test with 95% CI; *p<0.05; **p<0.01, ***p<0.001; ****p < 0.0001.

Individual patient blots for Figure 1F

Patient 1

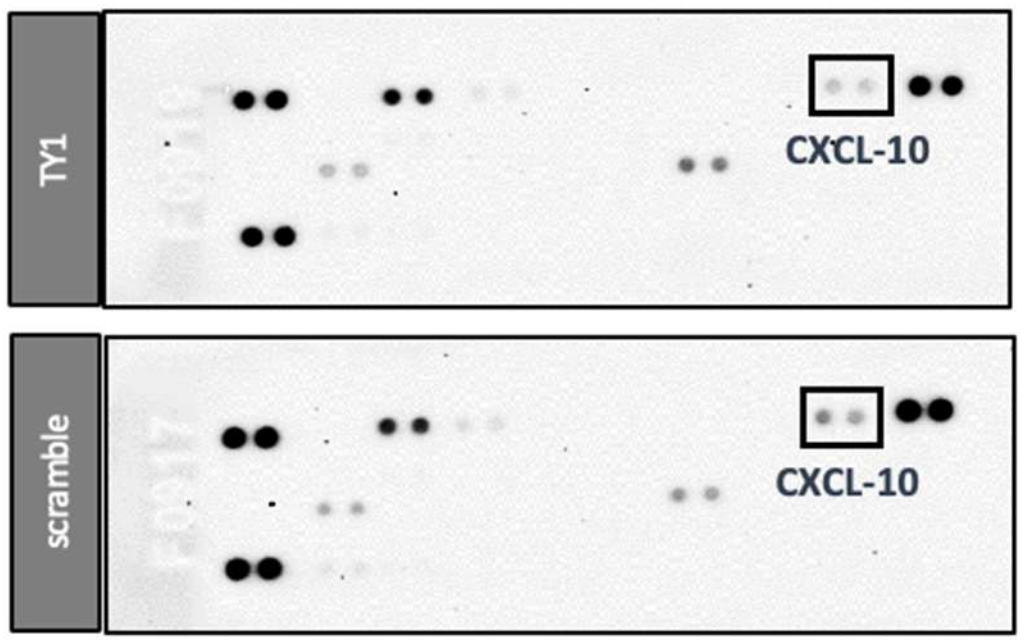

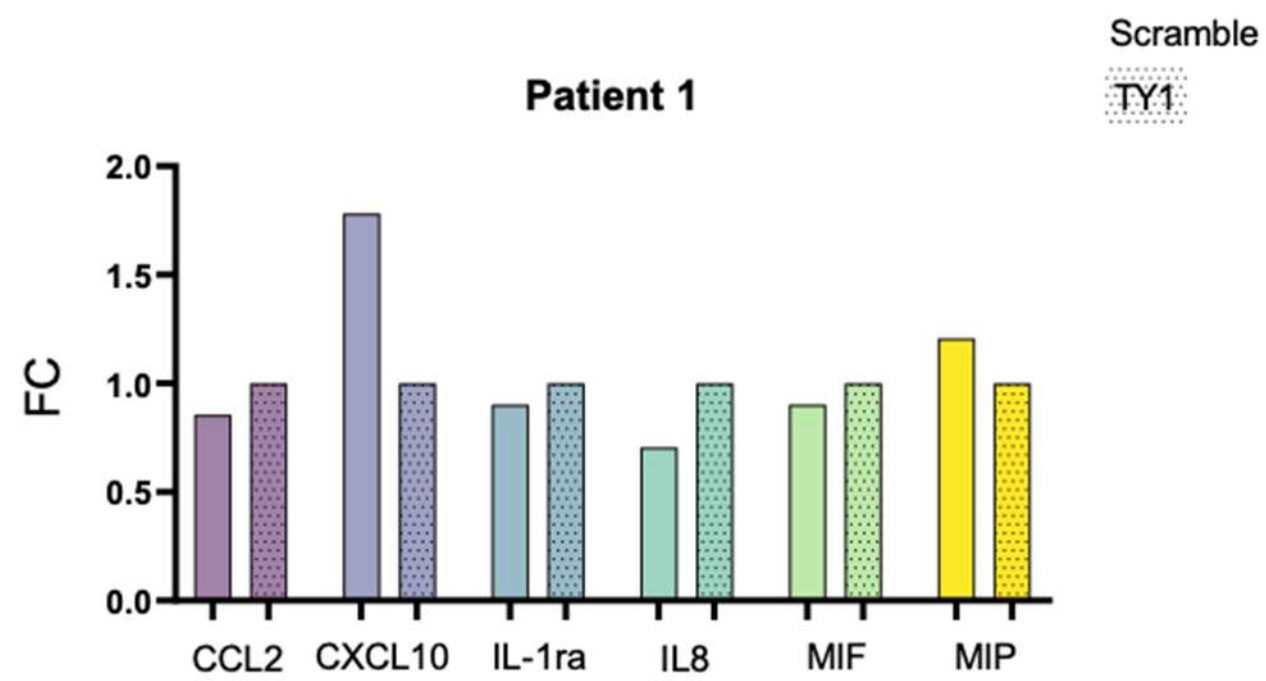

Patient 2

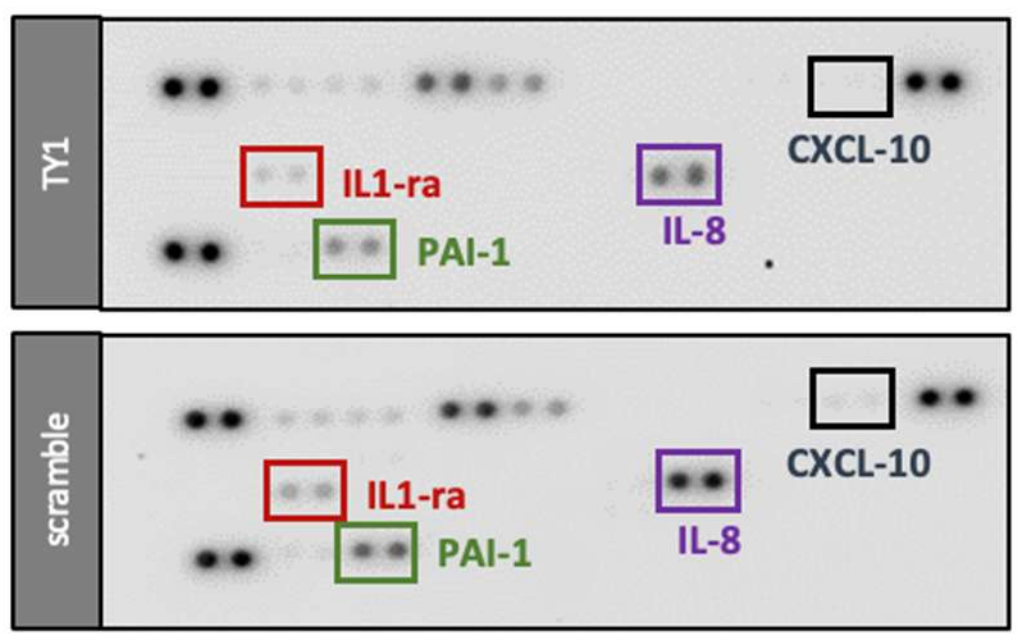

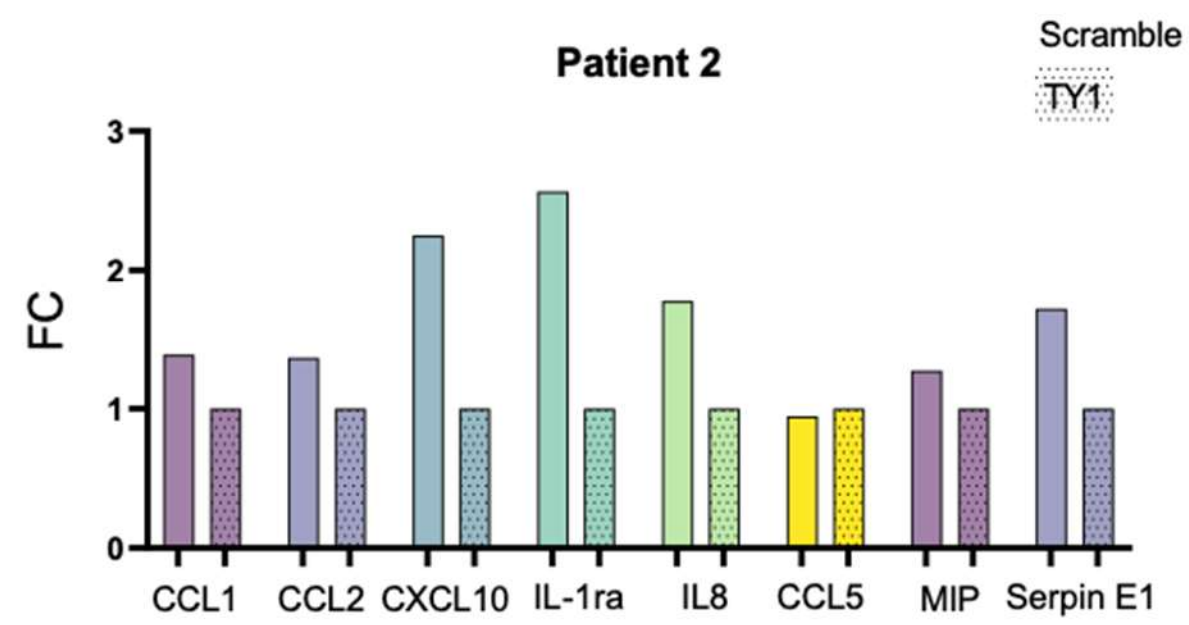

Patient 3

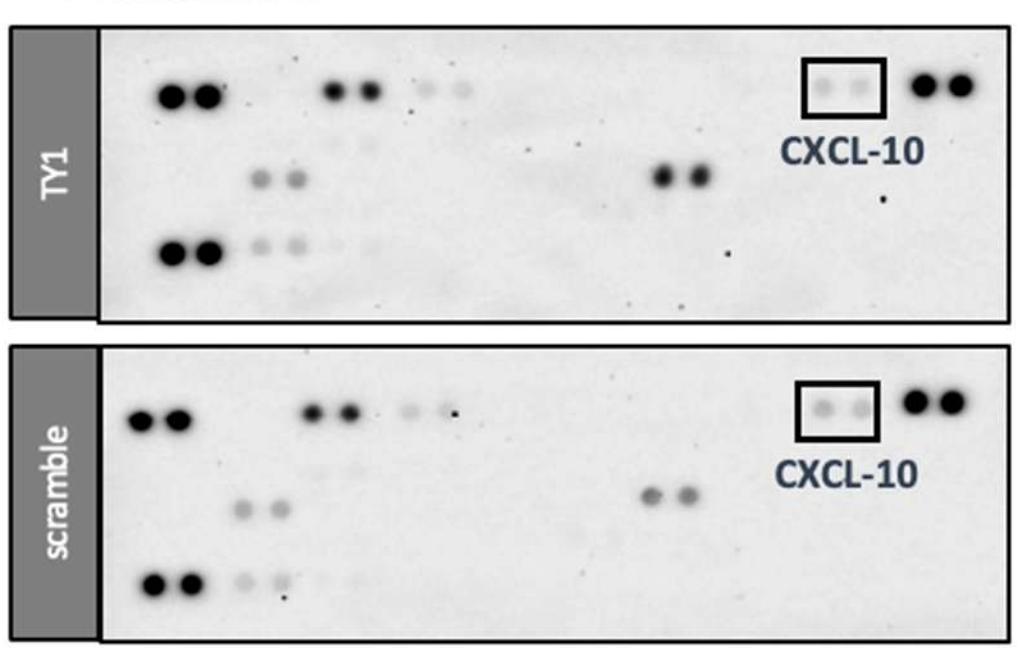

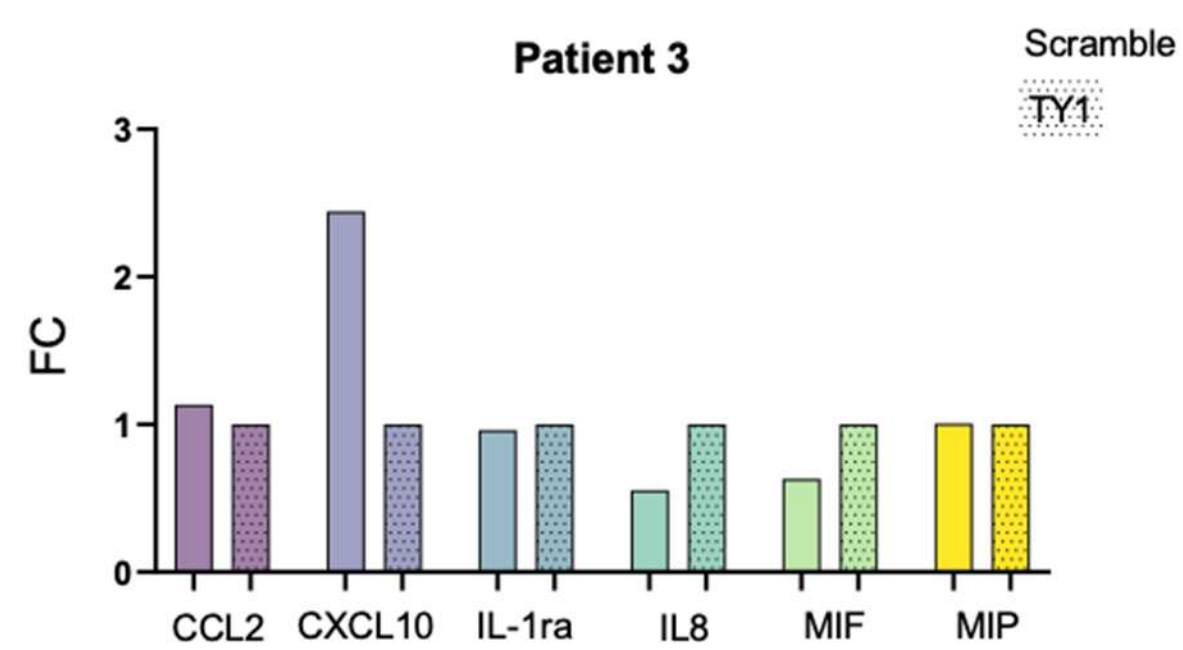

Supplement: Supplementary file 1 [file jciinsight-11-197716-s032.pdf]
