# Supplementary material for: Noncoding RNA TY1 reverses fibrosis in systemic sclerosis by attenuating the cGAS/STING pathway
Source: JCI Insight. 2026 Sep 8;11(17):e197716. doi: 10.1172/jci.insight.197716 (PMC13564078; doi:10.1172/jci.insight.197716)

Uncropped images for Figure 4E

cGAS

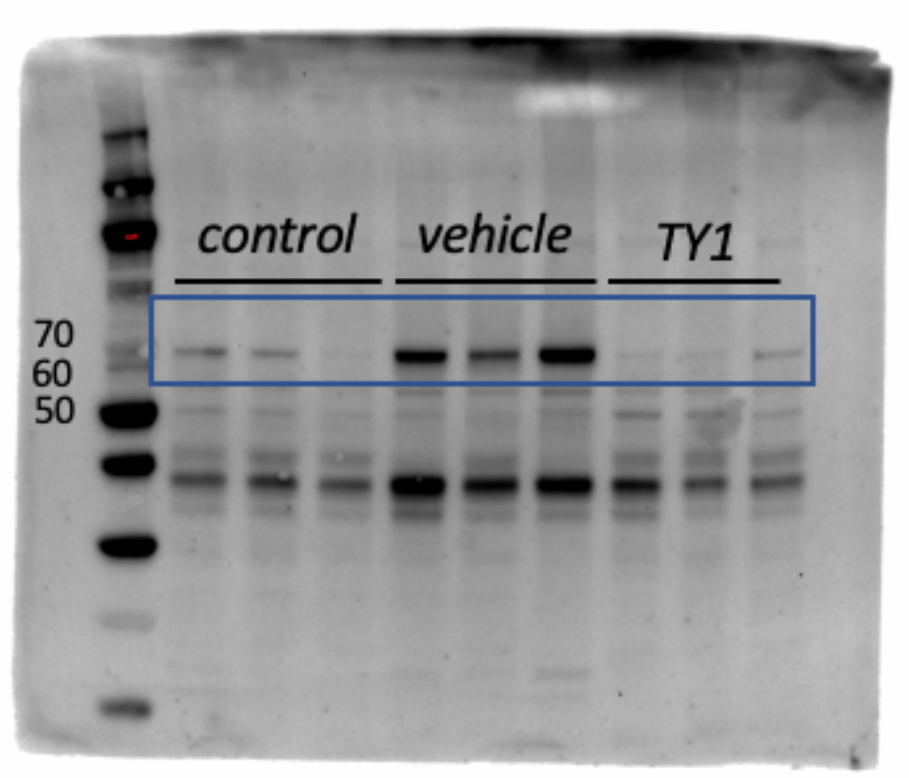

STING

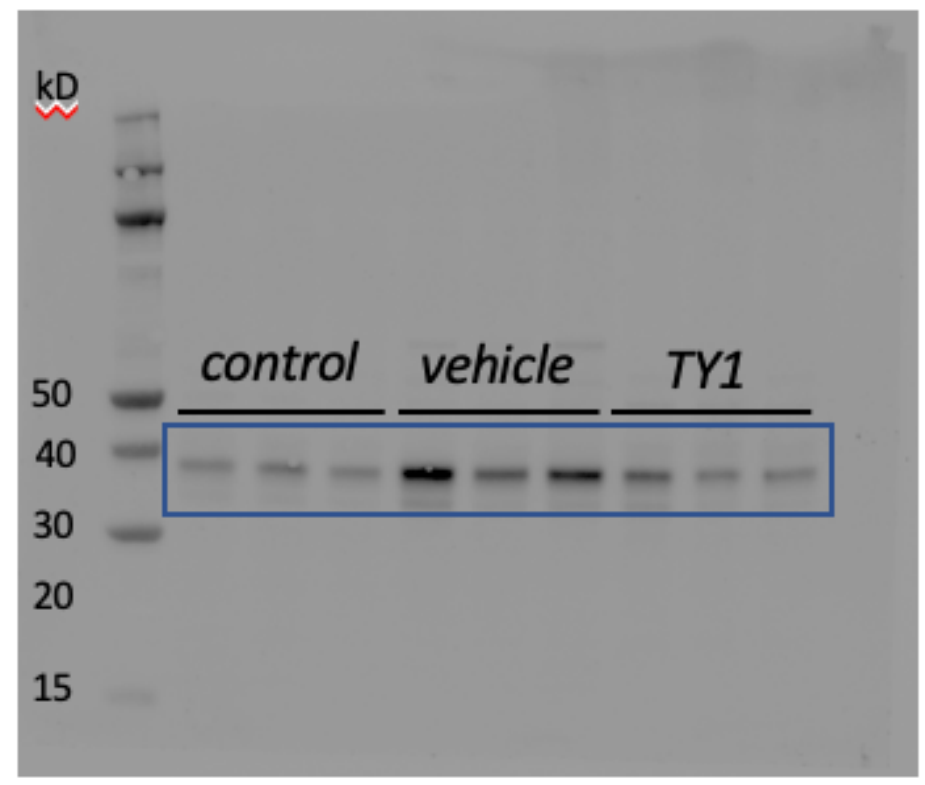

TBK1

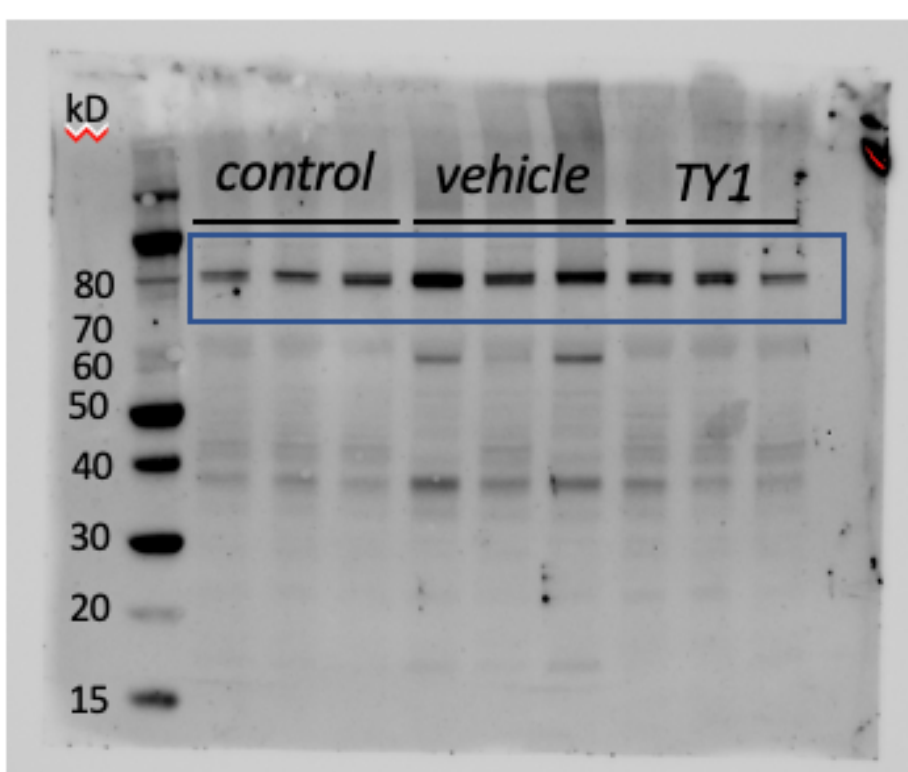

IRF3

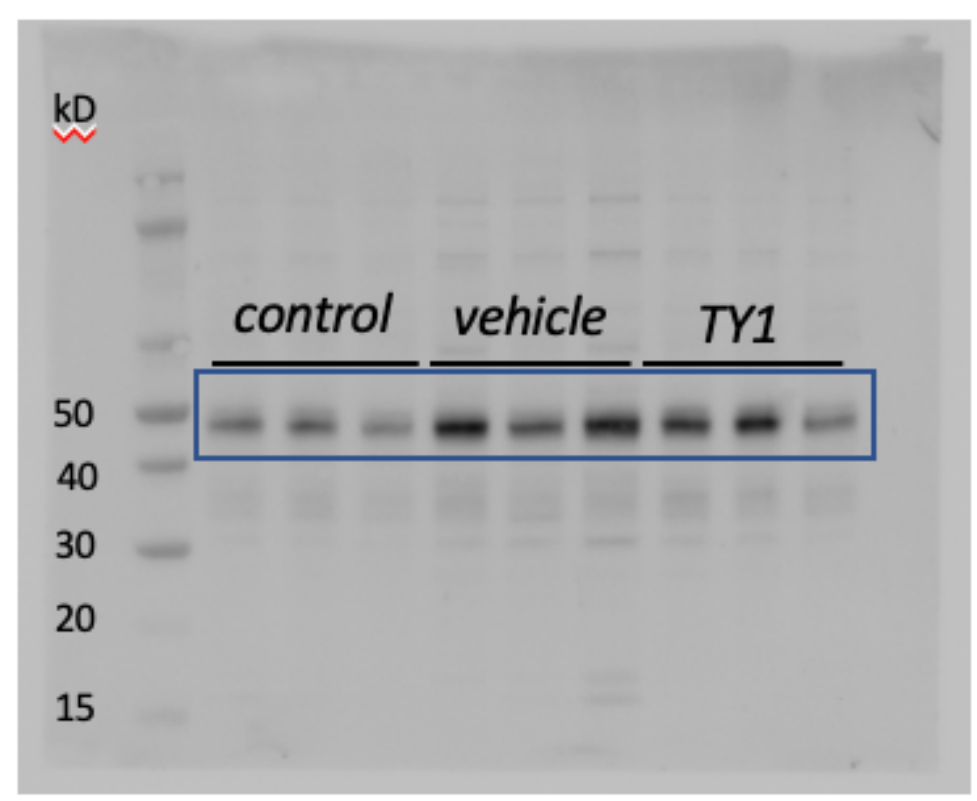

Pan-actin

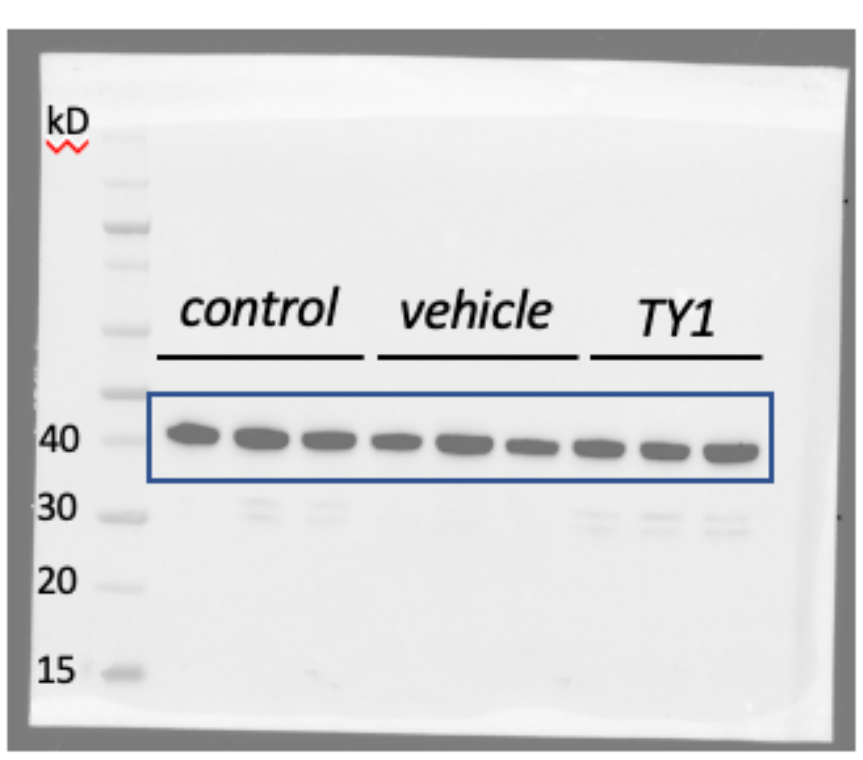

Trex1

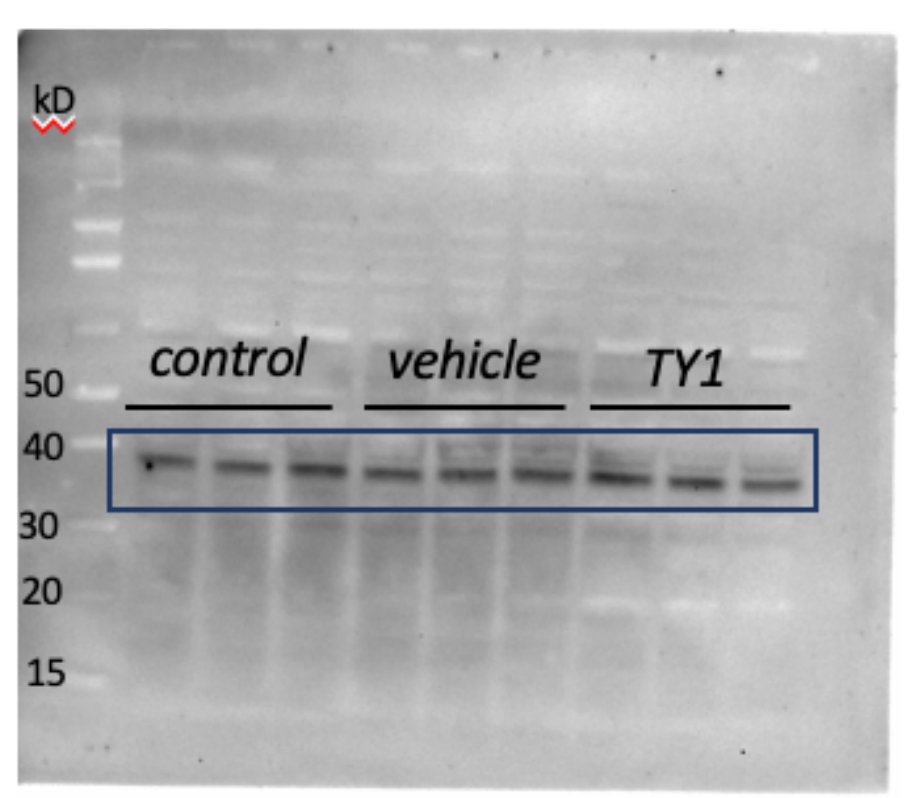

Uncropped images for Figure 1B

cGAS

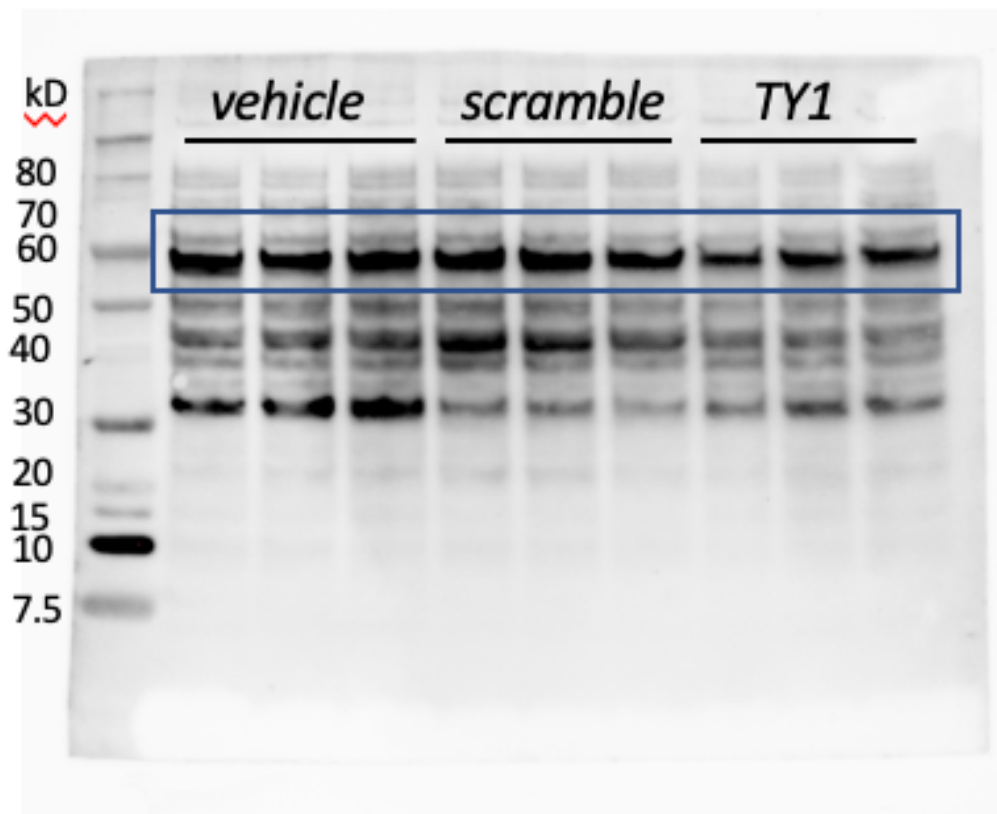

STING

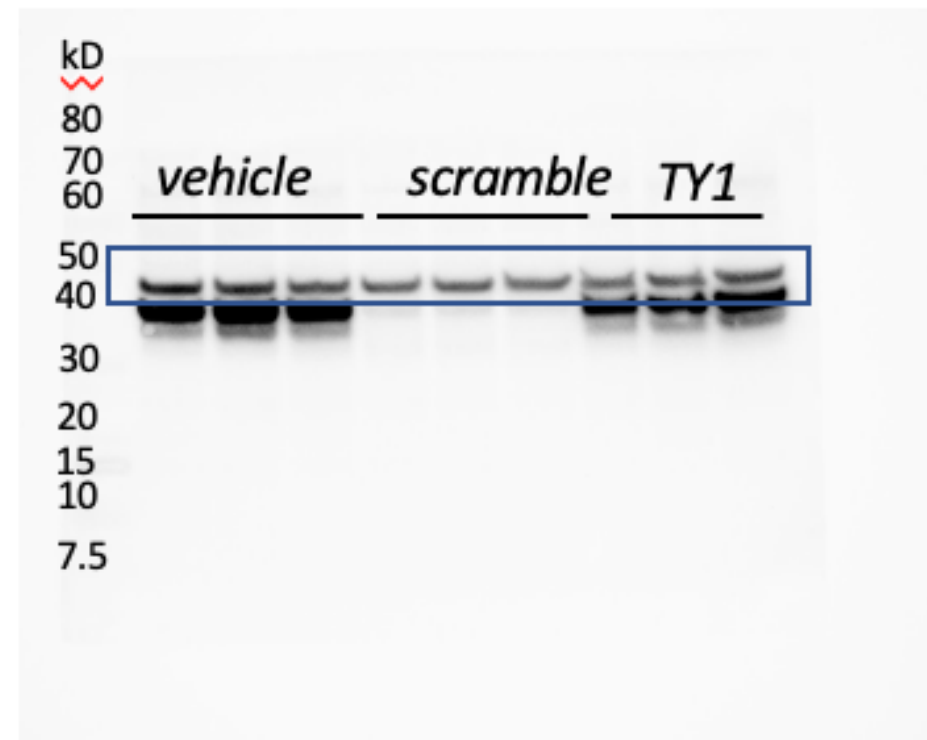

TBK1

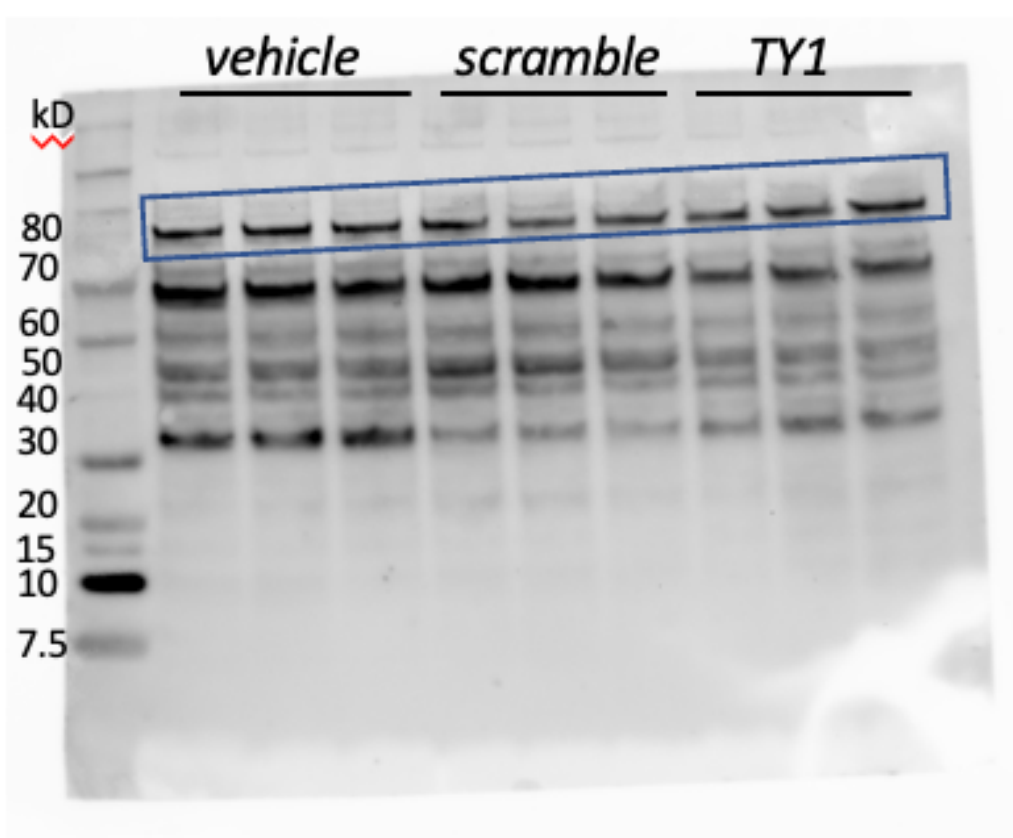

IRF3

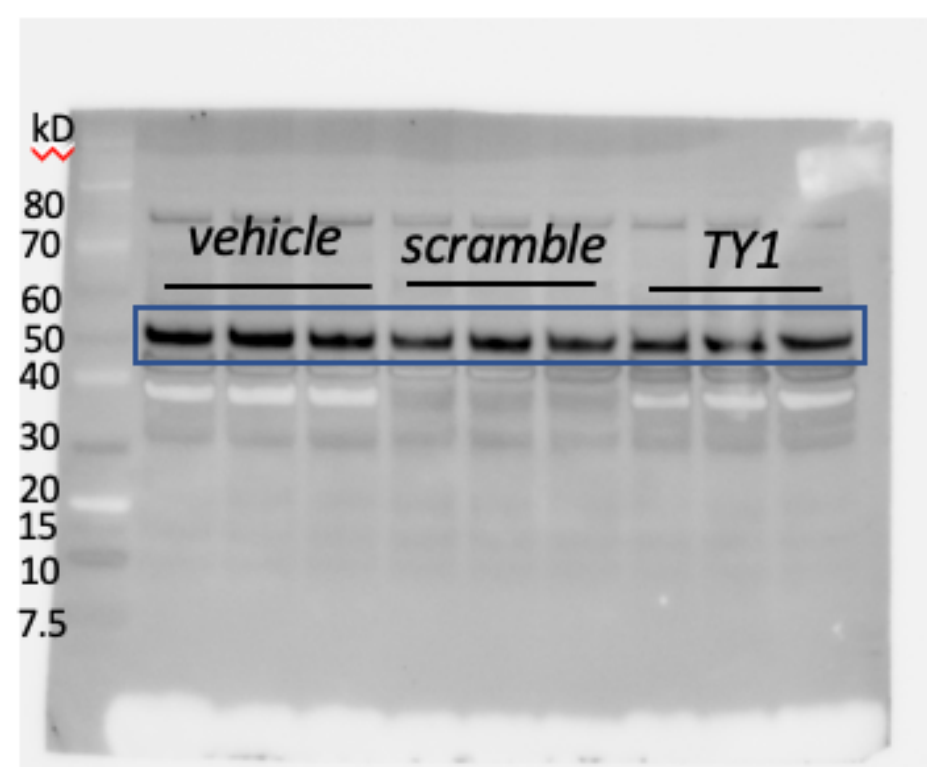

Pan-actin

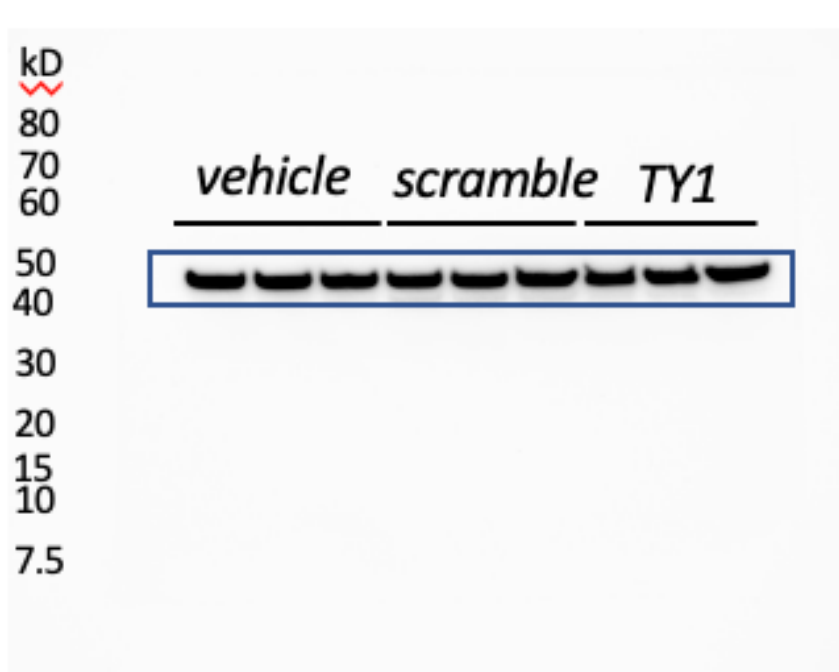

Supplement: Supplementary file 2 [file jciinsight-11-197716-s033.pdf]
